# FUBP1 promotes colorectal cancer stemness and metastasis via DVL1‐mediated activation of Wnt/β‐catenin signaling

**DOI:** 10.1002/1878-0261.13064

**Published:** 2021-07-29

**Authors:** Haofan Yin, Tianxiao Gao, Jinye Xie, Zhijian Huang, Xiaoyan Zhang, Fengyu Yang, Weiwei Qi, Zhonghan Yang, Ti Zhou, Guoquan Gao, Xia Yang

**Affiliations:** ^1^ Program of Molecular Medicine, Affiliated Guangzhou Women and Children’s Hospital Zhongshan School of Medicine Sun Yat‐Sen University Guangzhou China; ^2^ Sun Yat‐sen University Cancer Center State Key Laboratory of Oncology in South China Collaborative Innovation Center for Cancer Medicine Guangzhou China; ^3^ Department of Biochemistry Zhongshan School of Medicine SunYat‐sen University Guangzhou China; ^4^ Guangdong Engineering & Technology Research Center for Gene Manipulation and Biomacromolecular Products Sun Yat‐sen University Guangzhou China; ^5^ Guangdong Provincial Key Laboratory of Brain Function and Disease Sun Yat‐sen University Guangzhou China; ^6^ Key Laboratory of Tropical Disease Control (Sun Yat‐sen University) Ministry of Education Guangzhou China

**Keywords:** cancer stem cells, colorectal cancer, FUBP1

## Abstract

Distant metastasis is, unfortunately, the leading cause of death in colorectal cancer (CRC). Approximately 50% of CRC patients develop liver metastases, while 10–30% of patients develop pulmonary metastases. The occurrence of metastasis is considered to be almost exclusively driven by cancer stem cells (CSCs) formation. However, the key molecules that confer the transformation to stem cells in CRC, and subsequent metastasis, remain unclear. Far upstream element‐binding protein 1 (FUBP1), a transcriptional regulator of c‐Myc, was screened in CSCs of CRC by mass spectrometry and was examined by immunohistochemistry in a cohort of CRC tissues. FUBP1 was upregulated in 85% of *KRAS*‐mutant and 25% of wild‐type CRC patients. Further, whether in *KRAS*‐mutant or wild‐type patients, elevated FUBP1 was positively correlated with CRC lymph node metastasis and clinical stage, and negatively associated with overall survival. Overexpression of FUBP1 significantly enhanced CRC cell migration, invasion, tumor sphere formation, and CD133 and ALDH1 expression *in vitro*, and tumorigenicity *in vivo*. Mechanistically, FUBP1 promoted the initiation of CSCs by activating Wnt/β‐catenin signaling via directly binding to the promoter of *DVL1*, a potent activator of β‐catenin. Knockdown of *DVL1* significantly inhibited the transformation to stem cells in, as well as the tumorigenicity of, CRC. Activation of Wnt/β‐catenin signaling by *DVL1* increased pluripotent transcription factors, including c‐Myc, NANOG, and SOX2. Moreover, FUBP1 was upregulated at the post‐transcriptional level. Elevated FUBP1 levels in *KRAS* wild‐type CRC patients is due to the decrease in Smurf2, which promotes ubiquitin‐mediated degradation of FUBP1. In contrast, FUBP1 was upregulated in *KRAS*‐mutant patients through both inhibition of caspase 3‐dependent cleavage and decreased Smurf2. Our results demonstrate, for the first time, that FUBP1 is an oncogene, initiating the development of CSCs, as well as a new powerful endogenous Wnt‐signaling agonist that could provide an important prognostic factor and therapeutic target for metastasis in both *KRAS*‐mutant and wild‐type CRC.

## Graphical abstract

Distant metastasis represents the leading cause of death in colorectal cancer (CRC). Cancer stem cells (CSCs) formation is considered to drive metastasis in patients with CRC; however, the key molecules guiding CSC formation remain elusive. Here, we investigated the role of the FUBP1 oncogene and DVL1, a novel agonist of endogenous Wnt signaling in CSCs development. Our data indicate that FUBP1 acts in initiating the development of CSC and could serve as a prognostic factor, as well as a potential therapeutic target in both *KRAS*‐mutant and wild‐type patients with CRC metastasis.

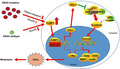

**Abbreviations**
ChIP: chromatin immunoprecipitation
CRC: colorectal cancer
CSCs: cancer stem cells
FUBP1: far upstream element‐binding protein 1
IHC: immunohistochemistry
Real‐time PCR: quantitative real‐time polymerase chain reaction

## 1 Introduction

Colorectal cancer (CRC) is the third lethality type of cancer worldwide. The incidence of tumor metastasis in CRC is increasing and often found before diagnose [1]. Most CRC patients die from recurrence and distant metastasis. Liver metastases occurs in approximately 50% of patients in CRC, while 10–30% of patients appear pulmonary metastases [2]. Metastasis is the main reason for the poor treatment and prognosis in CRC. Therefore, it is very urgent to elucidate the mechanism that leads to the metastasis of colorectal cancer and to find new molecular targets.

The occurrence of metastasis is considered to be almost exclusively driven by cancer stem cells (CSCs), which seeds and colonizes to distant organs [3, 4]. This small subpopulation of cells with tumor‐initiating property were found in colon, breast, head, and lung [5]. It was found that CD133^+^ CRC cells were more likely to metastasize than CD133^‐^ CRC cells in mice [6]. Additionally, the previous study showed that stem cell‐related markers CD133, CD44, and ALDH1 were more highly expressed in tissues with lymph node metastasis tissues than in CRC primary cancer [7]. Furthermore, a recent study found that in CSC profiles have a high prognostic impact for CRC patients, which further supports the hypothesis that CRC strongly links to the existence of changes in stem cell subpopulation [5, 8]. Consequently, CSCs‐targeted therapies might be an effective strategy to prevent metastasis of CRC. However, the key molecules that regulate colorectal cancer stem cells and subsequent metastasis remain unclear.

Single‐stranded DNA‐binding protein, far upstream element‐binding protein 1 (FUBP1), is highly expressed in various tumor tissues, such as renal cell carcinoma, squamous cell carcinoma, liver cancer, gastric cancer, breast cancer, non–small‐lung cancer, and Hodgkin's lymphoma [9]. FUBP1’s function includes promoting proliferation, inhibiting apoptosis of tumor cells by forming a complex with the far upstream element (FUSE) site to regulate gene expressions, inclusive of *c‐Myc*, *P21*, *P53*, etc [10, 11]. Our recent study revealed that elevated FUBP1 accelerated glycolysis leading to the proliferation of neuroblastoma cell through inhibiting the degradation of HIF1α by binding to the promoter of *VHL* [12]. Rabenhardt *et al*. [13] showed that FUBP1 could inhibit the mRNA transcription of cell cycle suppressor p21 on maintaining the self‐renewal of hematopoietic stem cells. Hwang *et al*. [14] found that FUBP1 regulated the selective splicing of LSD1 to change the maintenance of neural progenitor cells. Wesely *et al*. [15] revealed that knockout of FUBP1 delayed the differentiation of embryonic stem cells to mesoderm. These studies imply that FUBP1 is closely related to the maintenance and differentiation of stem cells. However, the expression and the exact role of FUBP1 in CRC and CRC‐related CSCs have not been investigated.

In this research, we aimed to identify the effects of FUBP1 on promoting colorectal cancer stemness and metastasis and the underlying mechanism.

## 2 Materials and methods

### 2.1 Human samples

54 cases of CRC tissue samples were collected from Sun Yat‐sen University Cancer Center. All patients' informed consent has been obtained before surgery, and the use of medical records and histological sections has also been approved by the ethics committee in SYUCC. The CRC tissue microarrays (HCol‐Ade180Sur‐08‐M‐088, 89 cases; HCol‐A150CS‐02‐M‐013, 75 cases) were purchased from Shanghai Outdo Biotech (Shanghai, China). All procedures were performed under consensus agreements and in accordance with the Chinese Ethical Review Committee. The study methodologies conformed to the standards set by the Declaration of Helsinki. The clinical and biological characteristics of the patients were described in Table S1.

### 2.2 Cell lines and culture

The human CRC cell lines (CaCO2, HCT‐116, SW48, LoVo, SW620) were obtained from the American Type Culture Collection (Manassas, USA). The normal intestinal epithelial cell lines (NCM460, CCD841) were provided by Professor Peng Huang, from Sun Yat‐sen University Cancer Center. Cell lines were authenticated by Celcook Biotech Co., Ltd. (Guangzhou, China). *KRAS* G13D SW48 was established by an improved CRISPR/Cas9‐mediated precise genetic modification by using 1 μm nonhomologous end‐joining (NHEJ) inhibitor Scr7 (Selleck, Houston, TX, USA, S7742).

### 2.3 Western blotting

Western blotting was performed according to a standard protocol, as described previously [16]. The total proteins were collected using SDS lysis buffer (Beyotime, Shanghai, China, P0013G), and protein concentrations were determined by Bicinchoninic Acid (KeyGen, Nanjing, China, KGP902). Nuclear extracts were obtained using the NE‐PER Nuclear and cytoplasmic extraction reagents (Thermo Scientific, Waltham, MA, USA, 78833) according to the manufacturer’s instructions. The following primary antibodies were used: FUBP1(ABE1330) from Merck Millipore (Boston, MA, USA); CD133 (#86781), ALDH1 (#54135), CD44 (#37259), p‐GSK3β (Ser9) (#9323), GSK3β (#9832), n‐p‐β‐catenin (Ser33/37/Thr41)(#8814), β‐catenin (#9582), Histone (4499), and c‐Myc (#13987) from Cell Signaling Technology (Boston, MA, USA); LGR5 (ab75732) and DVL1 (ab233003) from Abcam (Cambridge, UK); and β‐actin (A5441) from Sigma‐Aldrich (St. Louis, USA). HRP‐conjugated anti‐rabbit IgG (Cell Signaling Tech, #7074) and anti‐mouse IgG (Sigma‐Aldrich, AP308P) were used as secondary antibodies. Proteins were determined using ECL Plus Reagent (Millipore, WBKLS0100).

### 2.4 RNA isolation and RT‐qPCR

Gene expression validation by RT‐qPCR was performed as previously described [16]. The PCR primer sequences are listed in Table S2.

### 2.5 Immunohistochemistry staining

Immunohistochemistry was performed according to a standard protocol as described previously [16]. The slides were incubated with anti‐FUBP1, anti‐CD133, anti‐ALDH1, or anti DVL1 monoclonal antibodies at 4°C overnight. On the second day, the slides were treated with HRP‐conjugated secondary antibody and the antigen‐antibody complex was visualized by incubation with the DAB kit. Finally, all sections were counterstained with hematoxylin and photographed through a slide scanner (Axio Scan. Z1, ZEISS, Oberkochen, Germany). The degree of immunostaining was determined by the staining index (SI). The SI was calculated as the product of the grade of tumor cell proportions and the staining intensity score. The tumor cell proportions were graded as follows: 0, no positive tumor cells; 1, < 10% positive tumor cells; 2, 10–35% positive tumor cells; 3, 35–75% positive tumor cells; and 4, > 75% positive tumor cells. Staining intensity was scored as follows: 1, no staining; 2, weak staining (light yellow); 3, moderate staining (yellow‐brown); and 4, strong staining (brown). Accordingly, the protein expression as evaluated by SI has a possible score of 0, 1, 2, 3, 4, 6, 8, 9, 12, or 16. Samples with SI ≥ 6 were determined as high expression, and those with SI < 6 were determined as low expression.

### 2.6 Immunofluorescence staining

After fixed in 4% paraformaldehyde, cells were blocked with goat serum at 37 °C for 1 h. They were incubated with rabbit β‐catenin antibody at 4°C overnight and then were incubated with FITC conjugated goat anti‐rabbit IgG (Dako, Glostrup, Denmark, K500711) at 37 °C for 1 h after three times washing. Finally, the cell nucleus was stained with DAPI (Sigma‐Aldrich, Tokyo, Japan, D9542). Cells were visualized under Olympus BX51. Five randomly picked fields per slide were analyzed to determine the nuclear β‐catenin MOD, which represents the strength of staining signals as measured per positive pixels. MOD values for different groups of tissues were compared using the Student’s *t*‐test.

### 2.7 Migration and invasion assay

A total of 5 × 10^4^ cells in 200 μL serum‐free RPIM 1640 were seeded on cell culture inserts with 8 μm microporous filters (Corning, New York, NY, USA, 26616) coated with (invasion) or without (migration) Matrigel (BD Biosciences, Franklin Lakes, NJ, USA), and 500 μL of RPIM 1640 containing 10% FBS was added to the lower chamber. After being incubated for 48 h, the cells in the upper filters (inside the inserts) were removed, and the migrated or invaded cells in the lower filters (outside the inserts) were fixed in ethanol for 20 min, then stained with crystal violet for 10 min, and counted under a microscope. The number of migrated or invaded cells in 5 random optical fields (×100 magnification) of each filter from triplicate inserts was averaged.

### 2.8 Tumor sphere formation assay

1 × 10^3^ cells were seeded in 96‐well ultra‐low cluster plates (Corning, 3469) for 10 days. The tumor spheres were cultured in DMEM/F12 (Corning, R10‐092‐CV) serum‐free medium supplemented with 2% B27 (Thermo Scientific, Cat. No. 12587010), 20 ng·mL^−1^ epidermal growth factor (EGF, Beyotime, P5552), 20 ng·mL^−1^ basic fibroblast growth factor (bFGF, Beyotime, P6443), 5 μg·mL^−1^ insulin (Beyotime, P3376), and 0.4% BSA (Sigma‐Aldrich, Cat. No. A1933‐1G). After 10 days, the tumor spheres (tight, spherical, nonadherent masses > 50 µm in diameter) were counted, and their images were captured under an inverted microscope.

### 2.9 Luciferase reporter assay

Luciferase assay was performed using the Dual Luciferase Reporter Assay Kit (Promega, Madison, WI, USA) according to a standard protocol [12]. We defined *DVL1* promoter region spanning nucleotides −2100 to 0, relative to the transcription start site (TSS), as full‐length promoter. The human *DVL1* promoter region spanning nucleotides −2100 to 0 (relative to the TSS) generated by PCR amplification from LoVo cells were cloned into the NheI/BglII sites of pGL3‐basic luciferase reporter plasmid to generate DVL1 luciferase reporters. The human DVL1‐P1 promoter region spanning nucleotides −2100 to −1639, the human DVL1‐P2 promoter region spanning nucleotides −1638 to −1423, the human DVL1‐P3 promoter region spanning nucleotides −1422 to −1262, the human DVL1‐P4 promoter region spanning nucleotides −1261 to −626, and the human DVL1‐P5 promoter region spanning nucleotides −625 to 0 were generated by PCR amplification from LoVo cells. Respectively, these regions were cloned into the NheI/BglII sites of pGL4.26 luciferase reporter plasmid to generate luciferase reporters.

### 2.10 Cell sorting and flow cytometry

BD Influx Cell Sorter was used to sort out cells. To obtain the CD133^+^ALDH1^+^ cells, LoVo cells were labeled with primary anti‐CD133 (Thermo Scientific, 12‐1331‐82) monoclonal antibody. Aldefluor kit (STEMCELL Technologies, Vancouver, BC, Canada) was used to analyze the population of cells with high ALDH enzymatic activity. Before isolation, samples were subsequently washed and resuspended into single‐cell suspensions in phosphate‐buffered saline (PBS) for performing the separation. For the proportion of CD133^+^ALDH1^+^ cell detection, CRC cells were assessed and analyzed by flow cytometry using a CytoFLEX Flow Cytometer (Beckman Coulter, Pasadena, USA).

### 2.11 Chromatin immunoprecipitation (ChIP) assay

2 × 10^6^ Cells plated in a 100 mm culture dish were treated with 1% formaldehyde to cross‐link proteins to DNA. The cell lysates were sonicated to shear the DNA into 100–1000 bp lengths. Aliquots containing equal amounts of chromatin supernatants were incubated on a rocking bed at 4 °C overnight with either 1 μg FUBP1 antibody, or 1 μg IgG antibody as a negative control. Following reverse cross‐linking of protein–DNA complexes to free the DNA, PCR was carried out. The primers used in this study are listed in the Table S2.

### 2.12 Plasmids, retroviral infection, and transfection

All lentiviral vectors contained the puromycin resistance gene. Vectors encoding FUBP1 shRNAs were purchased from Hanbio Biotechnology Co., Ltd. (Shanghai, China). Vectors encoding DVL1 and c‐Myc were purchased from GeneChem Co., Ltd. (Shanghai, China). FUBP1 siRNA, DVL1 siRNA, c‐Myc siRNA, and control siRNA were purchased from RiboBio (Guangzhou, China). Plasmids encoding FUBP1 were purchased from Obio Technology Co., Ltd. (Shanghai, China); the lentiviruses were packaged, and cells were transduced and subjected to puromycin selection as previously described [16]. According to the manufacturer’s instructions, transfections were performed at approximately 60% confluency using Lipofectamine 3000. After 48 h, confirmation of interference or overexpression was carried out using real‐time quantitative PCR (RT‐qPCR) and western blotting. For sgRNA cloning, the CRISPR/Cas9 vector PX459 (Addgene, #62988) was digested with BbsI (Thermo Scientific, FD1014) and ligated with BbsI compatible annealed oligos. The sgRNA targeting the upstream sequence of *KRAS* exon2 (5′‐GCATTTTTCTTAAGCGTCGATGG‐3′) was designed using Optimized CRISPR Design. The homologous fragments used for introducing the point mutation were amplified by overlapping PCR and assembled into T vector pGM‐T (TianGen, Beijing, China, VT202) using In‐Fusion technology (Clontech, #639636, Mountain View, CA, USA). Next, the donor vector used for homology‐directed repair (HDR) was generated with the homologous fragment (amplified by overlapping PCR with attB1 at the ends) and pDONR (Thermo Scientific, #12536017) by BP reaction.

### 2.13 iTRAQ protein mass spectrometry

iTRAQ protein mass spectrometry was performed by PTM BIO Co., Ltd. (Hangzhou, China). The MaxQuant (version 1.4.1.2) software was used to analyze the raw data by GeneChem Co., Ltd.

### 2.14 Tumor xenograft

Male BALB/c nude mice (4‐week‐old, 16–18 g) were purchased from Beijing Vital River Laboratory Animal Technology Co., Ltd. (Beijing, China). All animals were kept in a specific pathogen‐free environment in this study. Based on a previously described standard protocol, the mice were randomly divided into the indicated groups [16]. SW48 or LoVo cells (1 × 10^6^, 1 × 10^5^, 1 × 10^4^, or 1 × 10^3^), stably transfected with FUBP1‐silenced, FUBP1 or vector, were inoculated into the inguinal folds of mice (*n* = 6 in each cell line per group). Also, CD133^+^ALDH1^+^ CSCs (1 × 10^5^, 1 × 10^4^, 1 × 10^3^, or 1 × 10^2^) sorted from LoVo cells, stably transfected with FUBP1‐silenced or vector, were inoculated into the inguinal folds of mice. Tumor volumes were measured with an external caliper and calculated using the equation (*L* × *W* ^2^)/2. At 28 days after inoculation, the mice were sacrificed, and the tumors were excised and subjected to pathologic examination. Sodium pentobarbital was used to anesthetize the mice to relieve pain. All procedures are related to animal feeding, and treatment and welfare were conducted in accordance with the Institutional Animal Care and Use Committee of Sun Yat‐sen University.

### 2.15 Statistical analysis

The variability of the data is presented as the SD (mean ± SD) and was assessed with Student’s *t*‐test between two groups. For multiple groups, significant differences were determined using one‐way ANOVA. The relationships between FUBP1 expression and clinicopathological characteristics were determined using the chi‐square test. Survival curves were plotted using the Kaplan–Meier method and compared using the log‐rank test. Survival data were evaluated using univariate and multivariate Cox regression analyses. Tumorigenic cell frequency (TIC) was calculated based on extreme limiting dilution analysis (ELDA) (http://bioinf.wehi.edu.au/software/elda/). Statistical significance was defined at *P* < 0.05.

## 3 Results

### 3.1 Elevated FUBP1 is associated with tumor progression in CRC

**Fig. 1:**
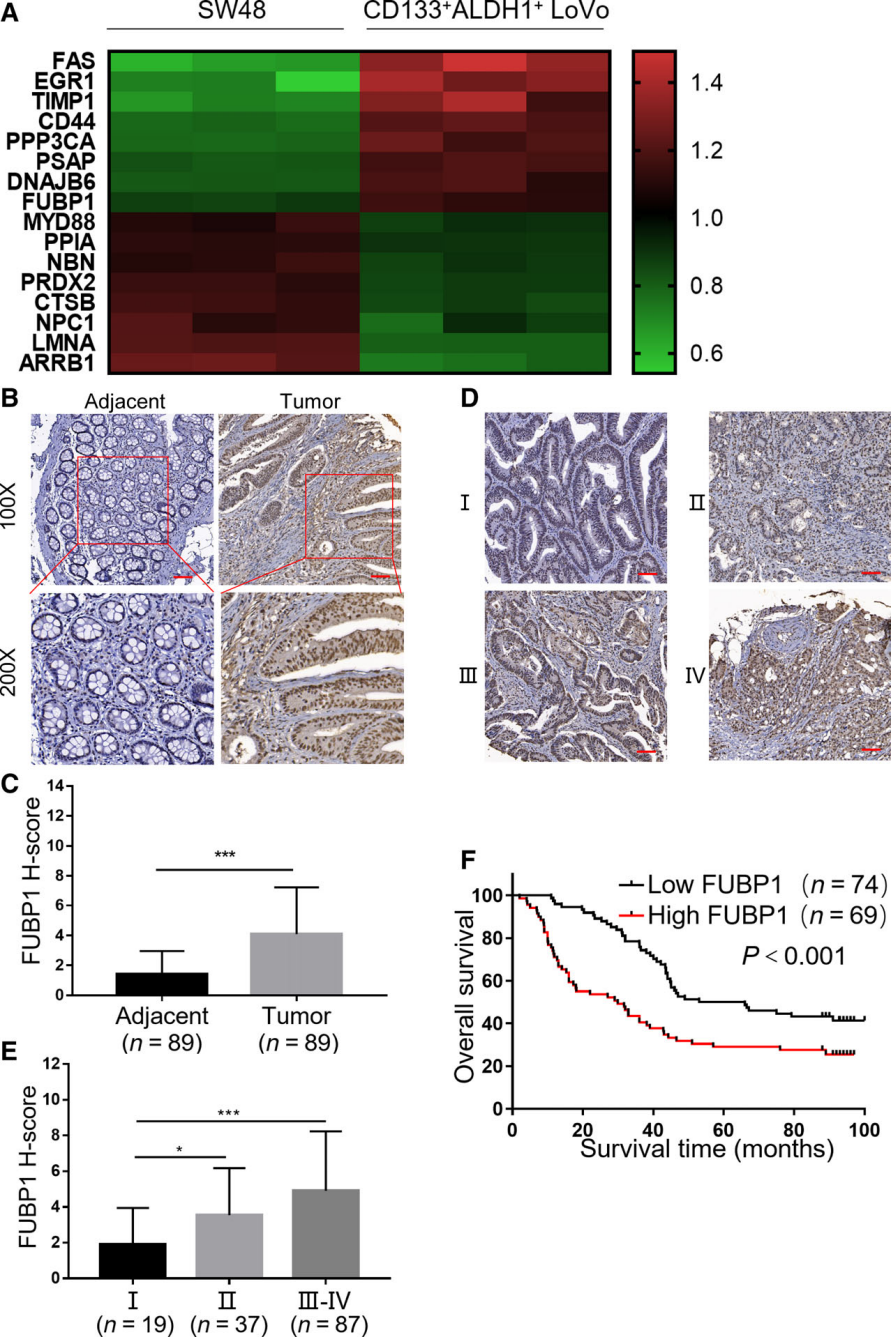
Elevated FUBP1 is associated with tumor progression in CRC. (A) Heat map illustrated overlap protein expression. This experiment was performed from triplicates for each condition. (B) Representative images of FUBP1 IHC staining of 89 adjacent specimens versus 89 CRC specimens in a CRC Tissue Microarray (top, 100× magnification; bottom, 200× magnification; HCol‐Ade180Sur‐08‐M‐088). Scale bar, 50 μm. (C) Statistical analysis of FUBP1 staining in adjacent specimens and CRC specimens. IHC quantification was performed using the staining intensity (SI). High FUBP1 expression was considered H‐Score ≥ 5; ***P < 0.001. (D) Representative images of FUBP1 IHC staining at different clinical stages (100× magnification). Scale bar, 50 μm. (E) Statistical analysis of FUBP1 staining at different clinical stages; *P < 0.05; ***P < 0.001. *P* values were determined by two‐tailed Student’s *t*‐test. Results were presented as mean ± SD. (F) Overall survival analysis of 89 CRC patients with low versus high FUBP1 expression; ***P < 0.001. *P* value was determined by log‐rank test.

LoVo cells derived from metastatic tumor tissue exhibited the strong ability of tumor sphere formation compared with SW48 cells derived from the primary site with low expression of CD133/ALDH (Fig. S1A–D). To explore the critical functional molecules in tumor stemness and aggressiveness, we sorted CD133^+^ALDH1^+^ LoVo cells which accounted for a 9.6% ratio in total LoVo cells. We then analyzed differential protein expression between CD133^+^ALDH1^+^ LoVo cell and SW48 cells by iTRAQ protein mass spectrometry (Fig. S1E). We used cell proliferation, cell movement, and cell cycle–related proteins database to predict the key proteins that regulated cell stemness, and the intersection proteins of three database were considered as the potential target proteins, and the results indicated that 16 proteins were tightly correlated with stemness (Fig. S1F). Among these proteins, transcription factor FUBP1 attracted our attention regarding its role in maintenance and differentiation of stem cells, while its connection with CRC was barely mentioned before (Fig. 1A).

Next, we verified the crucial role of FUBP1 in the progression of CRC. Impressively, compared with adjacent specimens (*H*‐Score = 1.411), the expression of FUBP1 was remarkably elevated in CRC specimens (*H*‐Score = 4.089; *P* < 0.001; Fig. 1B,C) in a CRC Tissue Microarray (Fig. S2A). Meanwhile, we retrospectively studied 143 CRC patients’ medical records and identified that FUBP1 expression increased along with the progression of CRC clinical stages (Fig. 1D,E). In addition, correlation analysis demonstrated that elevated FUBP1 positively associated with lymph node metastasis and advanced clinical stages in CRC (Table S1). Accordingly, the expression of FUBP1 was inversely correlated with overall survival (*P* < 0.001; Fig. 1F). The OS of the FUBP1 high expression group was even 30.25 months shorter than that of the low expression group (HR, 1.96; 95% CI, 1.291 to 2.974). Taken together, the upregulation of FUBP1 was closely relevant with CRC metastasis and might be a potential prognostic factor of CRC.

### 3.2 Elevated FUBP1 promotes CRC cell migration and invasion

**Fig. 2:**
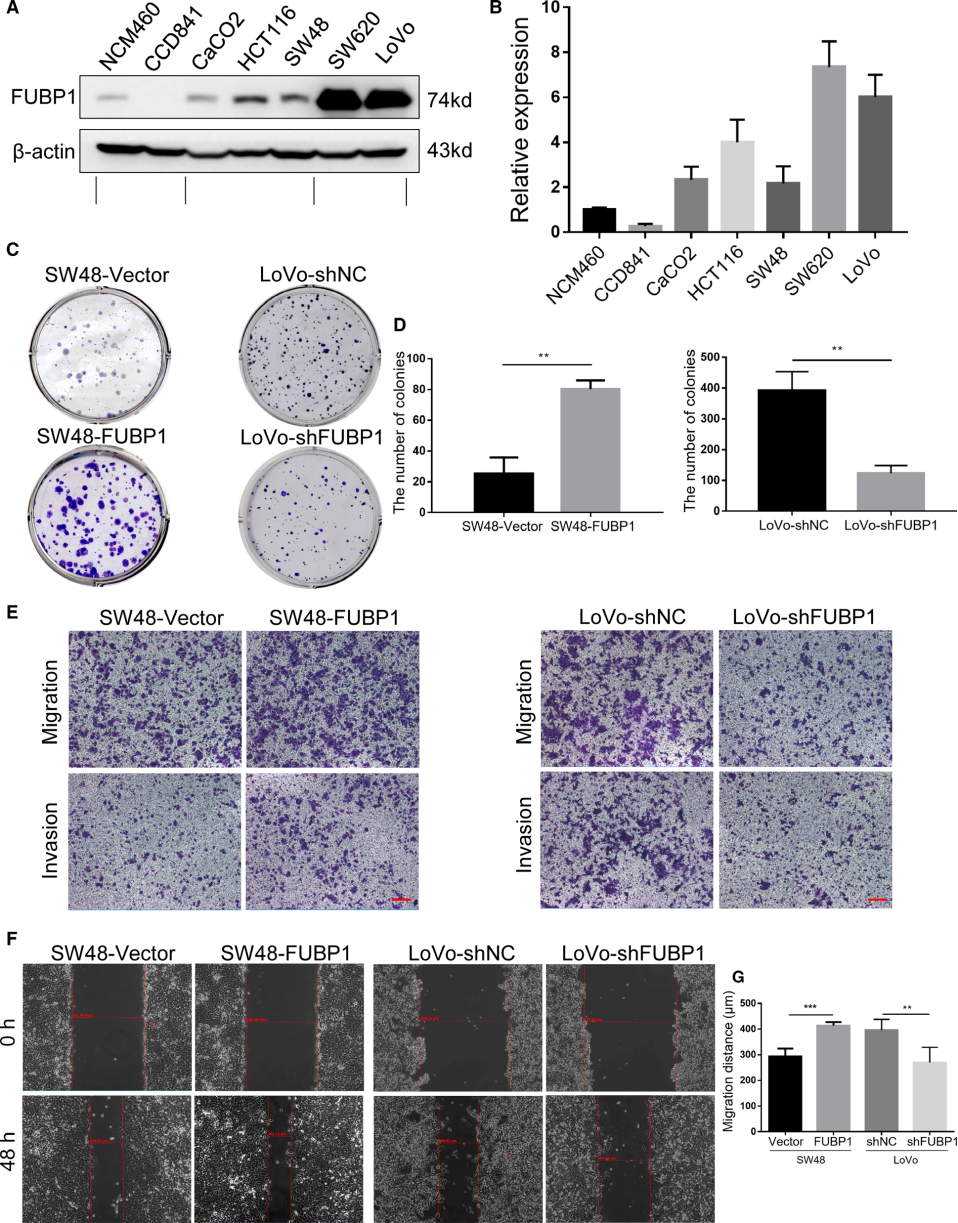
Elevated FUBP1 promotes CRC cell migration and invasion. (A) The protein levels of FUBP1 in CRC cells and normal intestinal epithelial cells by western blotting analysis. (B) The relative protein expression of FUBP1 was summarized as the mean ± SD of three independent experiments. (C) Representative images of colony formation in the indicated FUBP1‐transfected, FUBP1‐silenced, or vector control cells. (D) Statistical analysis of colony formation. **P < 0.01. (E) Representative images of transwell assays of migration and invasion by the indicated cells. Scale bar, 100 μm. (F) Representative images of wound‐healing assays by the indicated cells. (G) Statistical analysis of wound‐healing assays. **P < 0.01; ***P < 0.001. All bars represented the mean ± SD of three independent experiments. All *P* values were determined by two‐tailed Student’s *t*‐test.

Similar to the results in CRC tissues, FUBP1 expression was apparently augmented in CRC cells compared with normal intestinal epithelial cell lines (Fig. 2A,B). Moreover, CRC cells (LoVo, SW620) derived from tumor metastasis showed higher expression of FUBP1 than those (CaCO2, HCT116, SW48) derived from the primary site (Fig. 2A,B).

Furthermore, colony formation assay was performed to validate the cloning ability of CRC cells promoted by FUBP1. As shown in Fig. 2C,D, compared with the vector control cells, the numbers of the colony from FUBP1‐transfected SW48 cells increased, and conversely decreased in FUBP1‐silenced LoVo cells. Moreover, transwell and wound‐healing assay showed that the migration and invasion abilities were remarkably augmented in FUBP1‐transfected SW48 cells and were diminished in FUBP1 knockdown LoVo cells (Fig. 2E,G; Fig. S2B). Such stimulations on cell biological functions by FUBP1 were also observed in HCT116 and SW620 cells, which implied that the feature was not restricted to SW48 and LoVo cells (Fig. S3). Collectively, we concluded that the upregulation of FUBP1 promoted CRC cell migration and invasion.

### 3.3 Elevated FUBP1 promotes the stemness of CRC cells *in vitro*

**Fig. 3:**
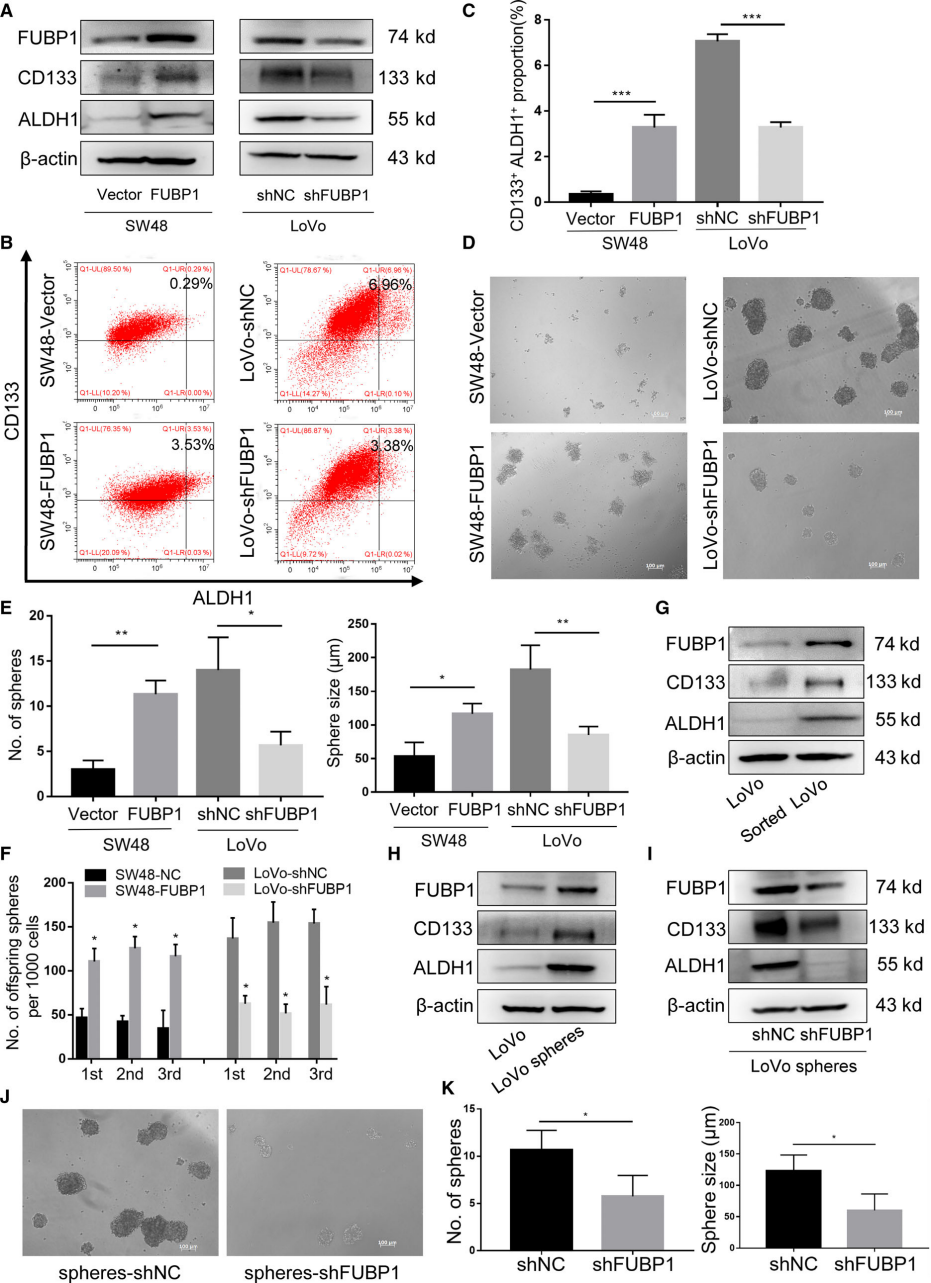
Elevated FUBP1 promotes the stemness of CRC cells *in vitro*. (A) Western blotting analysis of stemness‐related markers, CD133 and ALDH1, in the indicated cells. β‐Actin served as a loading control. (B) Flow cytometric analysis proportion of the coexpression of CD133‐PE and ALDH1‐FITC in the indicated cells. (C) Statistical analysis of the proportion of CD133^+^ALDH1^+^ cells. ***P < 0.001. (D) Representative images of tumor sphere formation after ten days in nonadherent cultures of the indicated cells. Scale bar, 100 μm. (E) Statistical analysis of sphere numbers and sizes after ten days in nonadherent cultures of the indicated cells. *P < 0.05; **P < 0.01. (F) The number of serially passaged spheroids was summarized as the mean ± SD of three independent experiments; *P < 0.05. (G) Western blotting analysis of FUBP1 in LoVo cells and CD133^+^ALDH1^+^ cells sorted from LoVo cells. (H) Western blotting analysis of FUBP1 in LoVo cells and LoVo spheres. (I) Western blotting analysis of CD133 and ALDH1 in FUBP1‐silenced cells and vector control cells, which were CD133^+^ALDH1^+^ cells sorted from LoVo cells. (J, K) Representative micrographs (J) and quantification (K) of tumor sphere formation by FUBP1‐silenced LoVo spheres and its control LoVo spheres. Scale bar, 100 μm. *P < 0.05. All bars represented the mean ± SD of three independent experiments. All *P* values were determined by two‐tailed Student’s *t*‐test.

CRC recurrence and distant metastasis arise from a subpopulation of CSCs, and FUBP1 expression was negatively associated with tumor differentiation status (Table S1), which implied that cell migration and invasion enhanced by FUBP1 might be attributed to the regulation of stemness. To explore the carcinogenic effect of FUBP1 in the stimulation of stemness in CRC cells, firstly, the protein levels of the stemness‐related markers, LGR5, CD133, ALDH1, and CD44, were examined between FUBP1 low expressing SW48 cells and FUBP1 high expressing LoVo cells. It showed that expression of stemness‐related markers, especially CD133 and ALDH1, was elevated in the LoVo cell (Fig. S1A). Meanwhile, western blotting results revealed that CD133 and ALDH1, were upregulated in FUBP1‐transfected SW48 cells, and conversely attenuated in FUBP1 knockdown LoVo cells (Fig. 3A). In addition, Flow cytometry results further indicated that overexpression of FUBP1 substantially increased the CD133^+^ALDH1^+^ percentage in SW48 cells from 0.34% to 3.27%, while knockdown of FUBP1 decreased the CD133^+^ALDH1^+^ percentage in LoVo cells from 7.06% to 3.28% (Fig. 3B,C). The expression of stemness‐related markers and the percentage of cancer stem cells were simultaneously upregulated in FUBP1‐transfected HCT116 cells, while conversely downregulated in FUBP1‐silenced SW620 cells (Fig. S4A–C).

Then, the tumor sphere formation assays were performed to inspect the influence of FUBP1 on the self‐renewal capability of spherogenic CRC cells. After 10‐day culture, the numbers and sizes of the formed spheres in the FUBP1‐transfected SW48 group were more remarkable than that of the vector control group, and the FUBP1‐silenced LoVo group exhibited the opposite effect (Fig. 3D,E). Such stimulations on tumor sphere formation by FUBP1 were also observed in HCT116 and SW620 cells (Fig. S4D,E). Furthermore, FUBP1‐transfected SW48 cells formed a more significant number of offspring spheres than the control, while FUBP1 knockdown cells formed less offspring spheres (Fig. 3F). Moreover, FUBP1 was responsible for the upregulation of pluripotent transcription factors c‐MYC [17], SOX2 [18], and NANOG [19]in CRC cells (Fig. S5A,B).

To further confirm that FUBP1 played a considerable role in CRC CSCs, we investigated the expression of FUBP1 in CD133^+^ALDH1^+^ cells sorted from LoVo cells by flow cytometer. As expected, CD133^+^ALDH1^+^ LoVo cells expressed higher level of FUBP1 than the LoVo cells (Fig. 3G). Similarly, LoVo spheres sorted by tumor sphere formation also showed higher FUBP1 levels than the LoVo cells (Fig. 3H). While CD133 and ALDH1 were significantly downregulated in FUBP1‐silenced LoVo spheres (Fig. 3I). Notably, knockdown of FUBP1 in LoVo spheres substantially reduced the numbers and sizes of the formed spheres, and decreased the migration and invasion abilities (Fig. 3J,K; Fig. S6). Collectively, these results indicated that the upregulation of FUBP1 promoted the stemness of CRC cells *in vitro*.

### 3.4 Elevated FUBP1 enhances the stemness and tumorigenicity of CRC cells *in vivo*

**Fig. 4:**
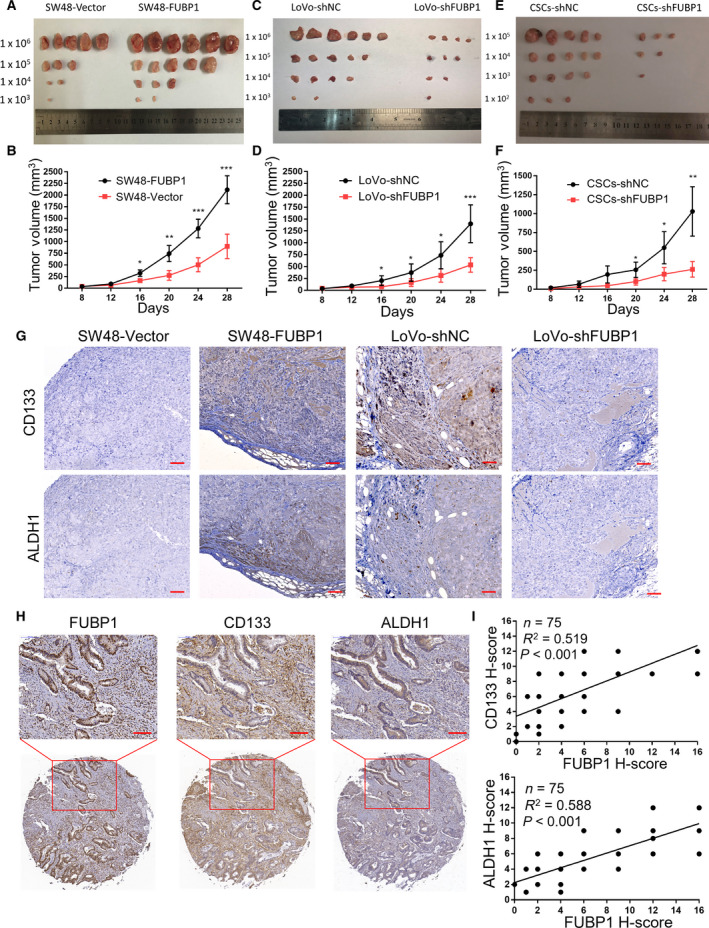
Elevated FUBP1 enhances the tumorigenicity of CRC cells *in vivo*. (A, C, E) Representative images of the tumors were shown. (A) Tumors formed by FUBP1‐transfected SW48 cells and vector control. (C) Tumors formed by FUBP1‐silenced LoVo cells and control LoVo cells. (E) Tumors formed by FUBP1‐silenced cells which were CD133^+^ALDH1^+^ cells sorted from LoVo cells and its control cells. Cells (1 × 10^6^, 1 × 10^5^, 1 × 10^4^, 1 × 10^3^, or 1 × 10^2^) were implanted into BALB/c nude mice (*n* = 6, per group). Tumor formation growth curves following implantation of the 1 × 10^6^ (B) (D)or 1 × 10^5^ (F) indicated cells. *P* values were determined by two‐tailed Student’s *t*‐test. (G) Representative images of IHC staining of CD133, ALDH1, and FUBP1 in tumor tissues of mice originated from the indicated cells. Scale bar, 50 μm. (H) Representative images of FUBP1, CD133, and ALDH1 IHC staining of CRC Tissue Microarrays (HCol‐A150CS‐02‐M‐013, 75 cases). Scale bar, 100 μm. (I) FUBP1 expression with the expression of CD133 and ALDH1 in 75 CRC patient specimens was determined by Pearson’s correlation analysis. All bars represented the mean ± SD of three independent experiments, and *, **, and *** denotes *P* < 0.05, *P* < 0.01, and *P* < 0.001, respectively.

To reveal the oncogenic effect of FUBP1 in promoting stemness *in vivo*, different amounts of CRC cells mixed with Matrigel were subcutaneously inoculated into the inguinal folds of BALB/c nude mice. The tumors formed by FUBP1‐transfected SW48 cells had a larger size and obvious tumorigenicity than those formed by vector control cells after implantation of 1 × 10^6^, 1 × 10^5^, 1 × 10^4^, or 1 × 10^3^ cells (Fig. 4A,B; Table S3). Conversely, FUBP1‐silenced LoVo cells formed smaller tumors and possessed blunt tumorigenicity (Fig. 4C,D; Table S3). Next, we use flow cytometry to isolate CD133^+^ALDH1^+^ LoVo cells from LoVo cell lines. Subsequently, knockdown the expression of FUBP1 in these CD133^+^ALDH1^+^ LoVo to observe tumorigenicity. Notably, the tumorigenicity of the sorted CD133^+^ALDH1^+^ LoVo cells was enhanced and fewer implantation of 1 × 10^5^, 1 × 10^4^, 1 × 10^3^, or 1 × 10^2^ cells were needed, meanwhile tumor size and tumorigenicity were quelled by FUBP1‐silence (Fig. 4E,F; Table S4). Western blotting and immunohistochemistry (IHC) results demonstrated that the expression of CD133 and ALDH1 in tumors originated from FUBP1‐transfected SW48 cells were increased, compared with that from vector control cells (Fig. 4G; Fig. S7). Notably, the expression of CD133 (*P* < 0.001; *R* ^2^ = 0.519) and ALDH1 (*P* < 0.001; *R* ^2^ = 0.588) in human CRC specimens were strongly positively correlated with FUBP1 in a CRC Tissue Microarray (Fig. 4H,I; Fig. S8). Therefore, we concluded that elevated FUBP1 enhanced the stemness and tumorigenicity of CRC cells *in vivo*.

### 3.5 Elevated FUBP1 mediates stemness through the activation of the Wnt/β‐catenin signaling

**Fig. 5:**
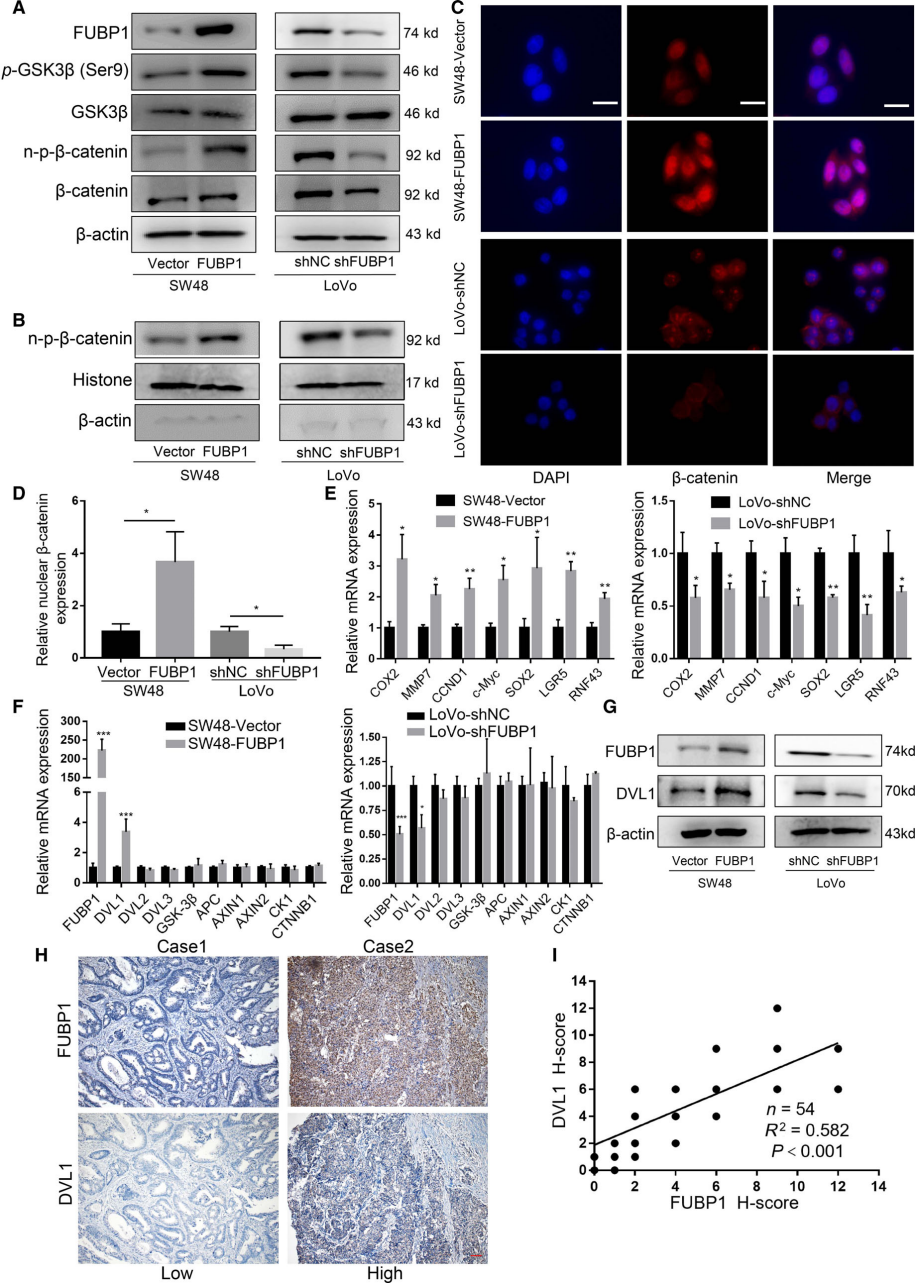
Elevated FUBP1 activates the Wnt/β‐catenin signaling. (A) Western blotting analysis of Wnt/β‐catenin signaling in the indicated FUBP1‐transfected, FUBP1‐silenced, and vector control cells. (B) Western blotting analysis of β‐catenin in the nuclear fractions of the indicated cells. (C, D) Immunofluorescence staining (C) and quantification (D) of nuclear β‐catenin expression in the indicated cells. Scale bar, 50 μm. *P < 0.05. *P* values were determined by two‐tailed Student’s *t*‐test. (E) The mRNA levels of β‐catenin downstream genes by real‐time PCR in the indicated cells. *P < 0.05; **P < 0.01. *P* values were determined by two‐tailed Student’s *t*‐test. (F) The mRNA levels of key scaffold molecules in the upstream of β‐catenin genes by real‐time PCR in the indicated cells. *P < 0.05; ***P < 0.001. *P* values were determined by two‐tailed Student’s *t*‐test. (G) Western blotting analysis of DVL1 in the indicated cells. (H) Immunohistochemistry staining of DVL1 in 54 CRC specimens which collected from Sun Yat‐sen University Cancer Center. Two representative cases are shown. Scale bar, 1 mm. (I) DVL1 expression with FUBP1 expression in CRC specimens was determined by Pearson’s correlation analysis; ***P < 0.001. All bars represented the mean ± SD of three independent experiments.

Wnt/β‐catenin signaling is well accepted to be involved in the stemness in CRC [8]. To probe the mechanism relevant with FUBP1‐mediated CRC stemness, we observed the Wnt/β‐catenin signaling in the indicated FUBP1‐silenced, FUBP1‐transfected, or control cells. As expected, FUBP1 positively regulated the phosphorylation level of GSK‐3β (Ser9) and nonphosphorylation levels of β‐catenin (Fig. 5A). Next, FUBP1 substantially increased the β‐catenin nuclear signals showed in nuclear extract and immunofluorescence assays whereas knockdown of FUBP1 reduced β‐catenin nuclear translocation (Fig. 5B–D). Meanwhile, upregulated mRNA transcription of the downstream targets of Wnt/β‐catenin signaling, including *COX2*, *MMP7*, *CCND1*, *c‐Myc*, *SOX2*, *LGR5*, and *RNF43*, was shown in FUBP1‐transfected cells showed, but decreased in FUBP1‐silenced cells (Fig. 5E). Moreover, overexpression of FUBP1 increased whereas knockdown of FUBP1 attenuated the transcriptional activation of TCF/LEF by TOP/FOP flash assays (Fig. S5C). Collectively, these data suggested that the Wnt/β‐catenin signaling pathway is activated by FUBP1 overexpression.

To further validated the targets of FUBP1 in the Wnt/β‐catenin pathway, real‐time PCR was used to detect the change of receptors and ligands which played essential roles in this pathway, including *LRP5*, *LRP6*, *FZD1*, *WNT3A*, and *WNT5A*, as FUBP1 had been proved to be an important transcription factor. However, none of these molecules are significantly altered (Fig. S9). Then, we detected mRNA levels of the critical scaffold molecules in the upstream of β‐catenin, including *DVL*, *GSK‐3β*, *APC*, *AXIN*, and *CK1*. Impressively, we found that *DVL1* mRNA levels were significantly upregulated by FUBP1, while other molecules remained unchanged (Fig. 5F). Moreover, FUBP1 increased the protein expression of DVL1 in CRC cells and tumor specimens, while silencing FUBP1 had the reverse effects (Fig. 5G; Fig. S7). Furthermore, the expression of FUBP1 and DVL1 was positively correlated in CRC tissues (*P* < 0.001; *R* ^2^ = 0.582; Fig. 5H,I). These results indicated that FUBP1 enhanced the Wnt/β‐catenin signaling through transcriptionally regulating DVL1.

### 3.6 FUBP1 upregulates DVL1 through direct binding to its promoter

**Fig. 6:**
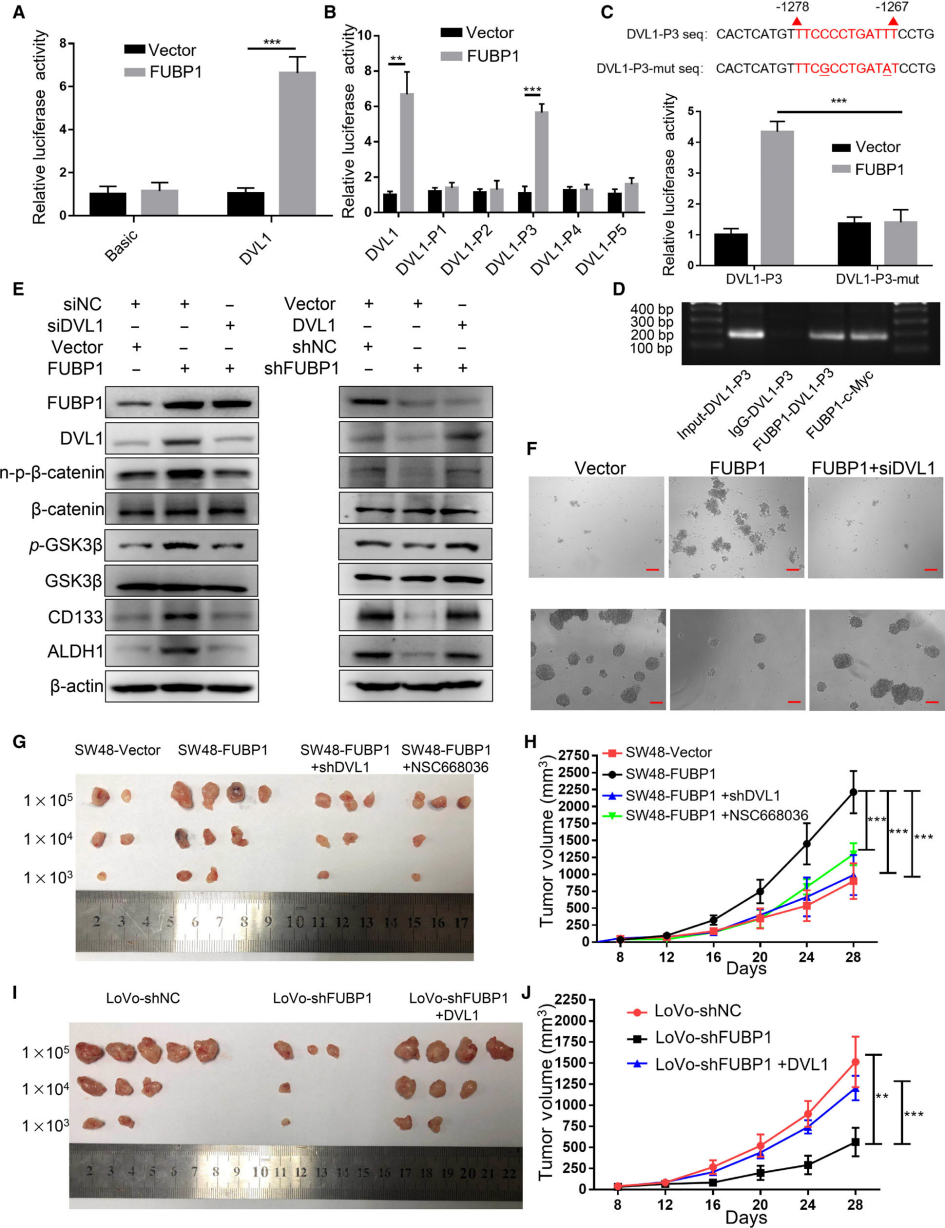
FUBP1 upregulates DVL1 through direct binding to its promoter. (A) Luciferase reporter assays of DVL1 promoter transcriptional activity. LoVo cells were infected with FUBP1‐overexpressing plasmid or empty vector plasmid, DVL1 promoter‐luciferase reporter plasmid, and Renilla luciferase plasmid for 48 h, followed by fluorescence detection. Renilla luciferase served as the transfection control. ***P < 0.001. (B) Luciferase reporter assays of DVL1 promoter truncation fragments included P1, P2, P3, P4, and P5 transcriptional activity. **P < 0.01; ***P < 0.001. (C) Luciferase reporter assays of mutant DVL1‐P3 promoter transcriptional activity. LoVo cells were infected with FUBP1‐overexpressing plasmid or empty vector plasmid, wild‐type DVL1‐P3 promoter reporter, and mutant reporter (C to G substitution and T to A substitution, underlined) for 48 h, followed by fluorescence detection. ***P < 0.001. (D) ChIP assays were performed to verify FUBP1 binding to the DVL1‐P3 promoter with c‐Myc group as positive control and IgG group as negative control. Lane 1: DVL1‐P3 PCR product from input DNA; Lane 2: DVL1‐P3 PCR product from immunoprecipitated by normal IgG; Lane 3: DVL1‐P3 PCR product derived from immunoprecipitation by anti‐FUBP1 antibody; Lane 4: c‐Myc promoter PCR product derived from immunoprecipitation by anti‐FUBP1 antibody. (E, F) Effects of FUBP1 on Wnt/β‐catenin signaling (E) and tumor sphere formation (F) were blocked after knockdown of DVL1 or recover after overexpression of DVL1. Scale bar, 100 μm. (G, H) The representative morphology (G) and tumor growth rate (H) were shown in SW48‐Vector, SW48‐FUBP1, SW48‐FUBP1+shDVL1, and SW48‐FUBP1+ NSC668036 xenograft models. (I, J) The representative morphology (I) and tumor growth rate (J) were shown in LoVo‐shNC, LoVo‐shFUBP1, and LoVo‐shFUBP1 + DVL1 xenograft models. **P < 0.01; ***P < 0.001. All bars represented the mean ± SD of three independent experiments. *P* values were determined by one‐way ANOVA.

To investigate how *DVL1* was transcriptionally regulated by FUBP1, promoter assays were performed. The luciferase reporter containing the full‐length *DVL1* promoter was transfected into LoVo cells, indicating that Elevated FUBP1 significantly increased *DVL1* promoter‐driven reporter activity (Fig. 6A). To identify the specific binding site, we constructed five truncation fragments of *DVL1* promoter, as indicated in Fig. S10A. Our results demonstrated that FUBP1 is bound to DVL1‐P3 fragments (Fig. 6B). Moreover, a potential binding site (TTCCCCTGATTT) in the DVL1‐P3 fragments was the same as the c‐Myc‐binding site [9]. The candidate binding site, TTCCCCTGATTT, was displayed in −1278 to −1267 region of *DVL1* promoter sequence (Fig. 6C). To identify whether FUBP1 could directly combine with this site, we constructed a mutation of DVL1‐P3 (C to G substitution and T to A substitution, underlined), and the mutation eliminated the FUBP1‐mediated increase in DVL1‐P3 promoter reporter activity. Moreover, we carried out Chromatin immunoprecipitation (ChIP) assays to investigate whether FUBP1 protein binds to DVL1‐P3 fragments at the chromosomal *DVL1* promoter region in LoVo cells. ChIP DNA enrichment was evaluated through PCR, which indicated that FUBP1 could directly bind to DVL1‐P3 fragments (Fig. 6D; Fig. S11A).

Then, we explored whether the stemness of CRC cells required DVL1 activation. Silencing DVL1 substantially decreased the expressions of stemness‐related markers (CD133 and ALDH1), the sphere‐forming ability, as well as Wnt/β‐catenin signaling in FUBP1‐transfected cells (Fig. 6E,F; Fig. S11B). Moreover, the increased abilities of CRC cell migration and invasion induced by overexpression of FUBP1 were significantly eliminated by knockdown of DVL1 (Fig. S10B,C). In addition, to verify the vital role of DVL1 in FUBP1‐induced tumorigenesis *in vivo*, cell line‐derived xenograft models were constructed by injecting SW48‐Vector, SW48‐FUBP1, SW48‐FUBP1 with DVL1 knockdown, and SW48‐FUBP1 with NSC668036 treatment (DVL inhibitor) [20]. It showed that SW48‐FUBP1 with DVL1 knockdown and treatment with NSC668036 in SW48‐FUBP1 xenografts inhibited the tumor volume and tumorigenicity significantly (Fig. 6G,H; Table S5). On the contrary, LoVo‐FUBP1 with DVL1 overexpression xenografts recovered the tumor volume and tumorigenicity compared with LoVo‐shFUBP1 xenografts (Fig. 6I,J; Table S6). Taken together, our results indicated that FUBP1 activated the Wnt/β‐catenin signaling to promote the stemness of CRC cells through upregulating DVL1 by direct binding to *DVL1*'s promoter.

### 3.7 FUBP1 is ubiquitinated by Smurf2 in CRC regardless of *KRAS* genotype

**Fig. 7:**
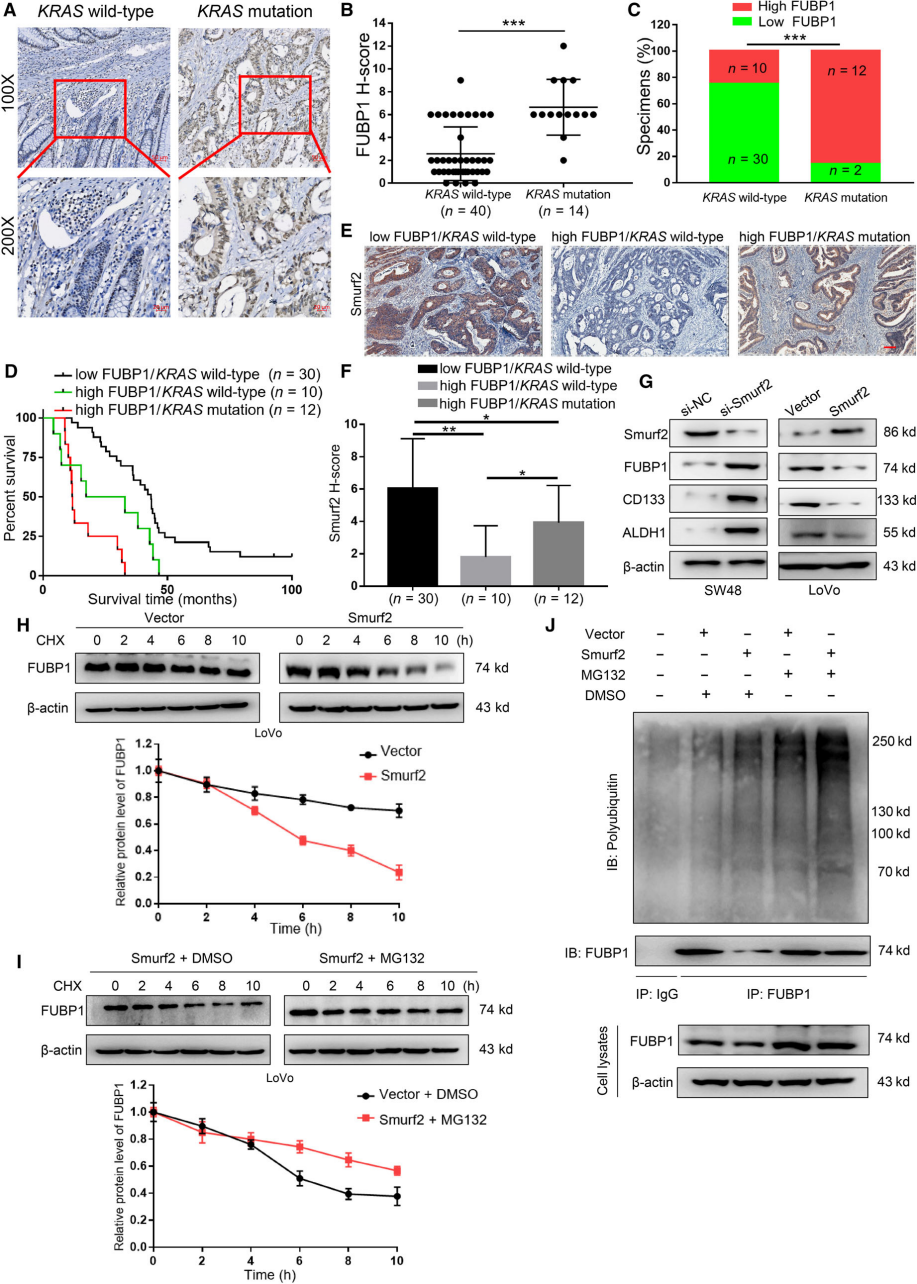
FUBP1 is ubiquitinated by Smurf2 in CRC regardless of *KRAS* genotype. (A) Representative images of FUBP1 IHC staining of 40 *KRAS* wild‐type CRC specimens versus 14 *KRAS* mutation CRC specimens collected from Sun Yat‐sen University Cancer Center (top, 100× magnification; bottom, 200× magnification). Scale bar, 50 μm. (B, C) Statistical analysis of the 54 CRC specimens showing low or high FUBP1 expression relative to *KRAS* wild‐type or *KRAS* mutation. ***P < 0.001. (D) Overall survival analysis of 52 CRC patients which divided into three groups: *KRAS* wild‐type and low FUBP1 expression (*n* = 30); *KRAS* wild‐type and high FUBP1 expression (*n* = 10); *KRAS* mutation and high FUBP1 expression (*n* = 12). (E) Representative images of Smurf2 IHC staining in three groups according to FUBP1 expression (high or low) and *KRAS* mutation or wild‐type. Scale bar, 100 μm. (F) Statistical analysis of Smurf2 staining in three groups; *P < 0.05; **P < 0.01. (G) Western blotting analysis of FUBP1, CD133, and ALDH1 in the indicated cells. β‐Actin served as a loading control. (H, I) Cycloheximide chase assay of FUBP1. (H) LoVo cells transiently transfected with vector or Smurf2 plasmids were treated with cycloheximide (CHX, 10 μg·mL^−1^) for indicated time points and then collected for western blotting analysis. (I) LoVo cells transfected with Smurf2 plasmid were treated with DMSO or MG132 (10 μmol·L^−1^) in the presence of CHX (10 μg·mL^−1^) for indicated time points and then collected for western blotting analysis. (J) LoVo cells transiently transfected with Vector or Smurf2 plasmids were treated with DMSO or MG132 (10 μmol·L^−1^) for 12 h, and then immunoprecipitated with an FUBP1 antibody. All bars represented the mean ± SD of three independent experiments. *P* values were determined by one‐way ANOVA.

The above data suggested that FUBP1 played a crucial role in the metastasis and stemness of CRC. The critical question arose up that the intrinsic mechanism by which FUBP1 was upregulated in CRC. *KRAS*‐mutant are one of the most frequent alterations, occurring in 30–50% of CRC cases. Further investigation of 54 CRC specimens identified that FUBP1 was highly expressed in *KRAS*‐mutant CRC specimens (*n* = 14) compared with *KRAS* wild‐type specimens (*n* = 40; *P* < 0.001; Fig. 7A,B). The proportion of high FUBP1 expression (*H*‐Score ≥ 6) in the *KRAS*‐mutant group (85.71%, 12/14) was higher than that of the *KRAS* wild‐type group (25.00%, 10/40; Fig. 7C). Moreover, *KRAS*‐mutant significantly increased the protein expression of FUBP1, while knockdown *KRAS* exhibited the reverse effects in CRC cells (Fig. S12C). Unexpectedly, the RNA level of FUBP1 in the tumor tissues did not changed compared with adjacent tissues by analyzing the TCGA‐COAD database and remain consistent in the LoVo cell compared with the SW48 cell (Fig. S12A,B).

Interestingly, we found that the proportion of high FUBP1/*KRAS* wild‐type specimen remained as 18.52% of total CRC pathological samples (10/54; Fig. 7C). The survival outcomes of patients with high FUBP1 expression in both *KRAS* wild‐type (HR, 2.369; 95%CI, 0.923 to 6.080; *P* = 0.013) and *KRAS*‐mutant (HR, 5.201; 95%CI, 1.612 to 16.78; *P* < 0.001) were much poorer compared with simultaneous low FUBP1/*KRAS* wild‐type patients (Fig. 7D), while the survival outcomes of patients had no statistical difference between high FUBP1/*KRAS* wild‐type and high FUBP1/*KRAS* mutation groups (*P* = 0.066; Fig. 7D). FUBP1 RNA levels were not statistically different in CRC specimens between *KRAS* mutation, *KRAS* wild‐type, and adjacent CRC specimens (Fig. S12D). These results suggest that FUBP1 might be post‐transcriptionally regulated in CRC regardless of *KRAS* mutation.

Recently, studies showed that Smurf2 was responsible for the ubiquitination of FUBP1 [21]. Excitingly, lower expression of Smurf2 was found only in the high FUBP1/*KRAS* wild‐type group (Fig. 7E,F). Silencing Smurf2 substantially increased the expression of FUBP1 and the stemness‐related markers, CD133 and ALDH1, conversely decreased in Smurf2 overexpressed cells (Fig. 7G). Consistently, our results revealed that Smurf2 substantially shorten protein half‐lives of FUBP1 via proteasomal degradation pathway (Fig. 7H,I). Furthermore, MG132 treatment with ectopically expressed Smurf2 significantly enhance FUBP1 polyubiquitination levels (Fig. 7J). These results demonstrated that the FUBP1 level was post‐transcriptionally by Smurf2 in *KRAS* mutation and part of *KRAS* wild‐type CRC.

### 3.8 FUBP1 is also upregulated by caspase 3 inactivation driven by *KRAS* signaling in *KRAS* mutation CRC

Moreover, previous research demonstrated that FUBP1 was the hydrolyzed substrate of caspase 3 [10], which is mainly suppressed by the antiapoptosis AKT and ERK signaling. *KRAS* mutation, which directly activates ERK and AKT pathways, has been recently linked to CSCs‐like phenotypes [22]. Therefore, we hypothesized that inhibition of caspase 3 activity through activating the antiapoptosis AKT and ERK signaling mediated by *KRAS* mutation also contributed to the abnormal increase in FUBP1.

**Fig. 8:**
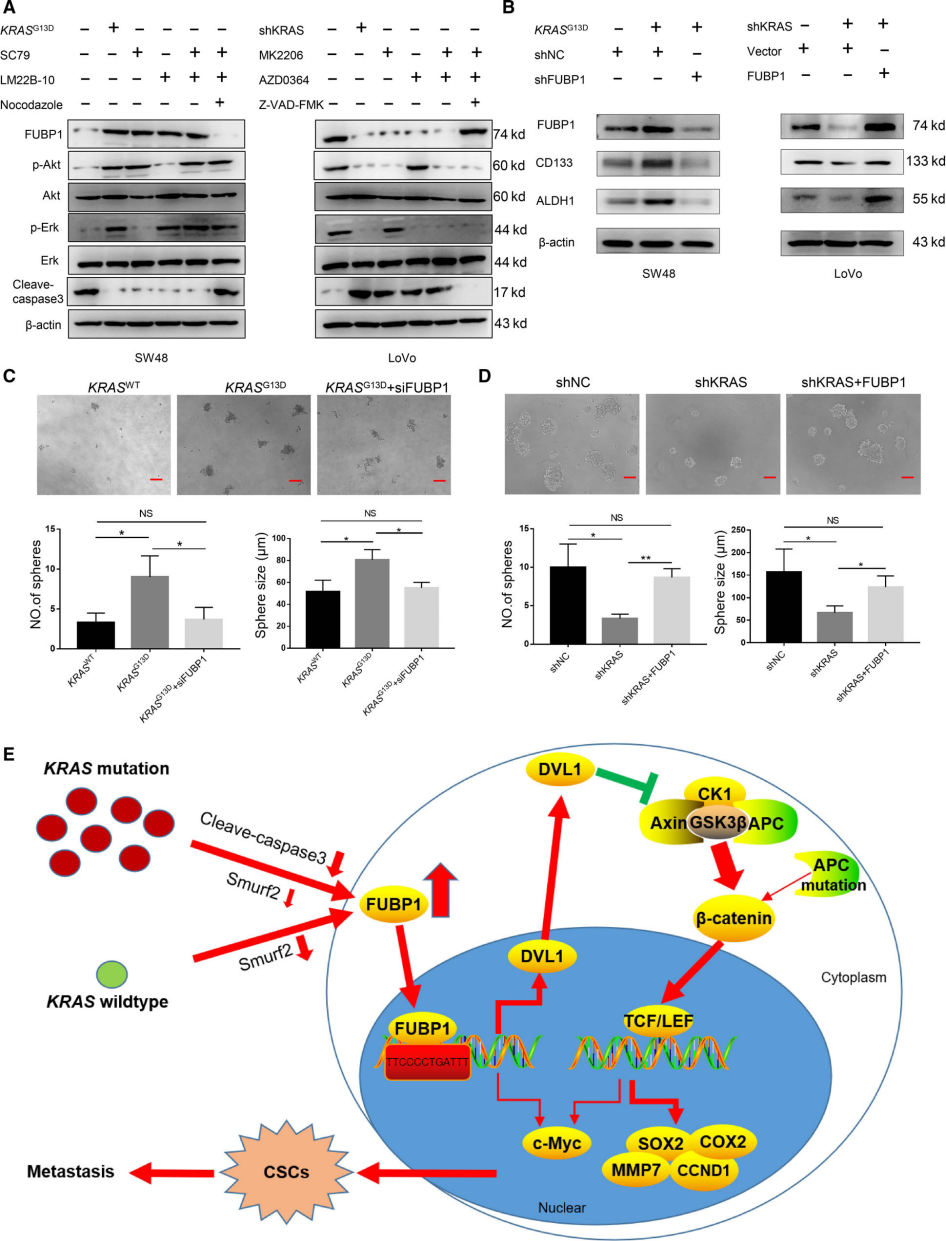
FUBP1 is also upregulated by caspase 3 inactivation driven by *KRAS* signaling in *KRAS* mutation CRC. (A)Western blotting analysis of FUBP1, phosphorylated AKT, total AKT, phosphorylated ERK, total ERK, and cleaved caspase 3 in SW48 cells with *KRAS* mutation or AKT, ERK, and apoptosis activators and in LoVo cells with KRAS‐silencing or AKT, ERK, and apoptosis inhibitors. (B–D) Effects of *KRAS* mutation on stem‐related markers (B) and tumor sphere formation (C, D) were blocked after knockdown of FUBP1 or recover after overexpression of FUBP1. *P < 0.05; **P < 0.01. Scale bar, 100 μm. (E)The schematic overview of the potential mechanism involved in FUBP1 overexpression promotes the stemness of CRC cells. All bars represented the mean ± SD of three independent experiments. *P* values were determined by one‐way ANOVA.

Next, we observed the protein expression induction of FUBP1 in SW48 treated with SC79 (AKT activator), LM22B‐10 (ERK activator), Nocodazole (apoptosis activator) or *KRAS* mutation, along with FUBP1 expression in LoVo treated with MK2206 (AKT inhibitor), AZD0364 (ERK inhibitor), Z‐VAD‐FMK (apoptosis inhibitor), or KRAS knockdown (Fig. 8A). It showed that AKT and ERK activators increased the expression of FUBP1 in SW48 almost close to that of SW48 *KRAS* ^G13D^, while apoptosis agonist could block this induction. On the contrary, AKT and ERK inhibitors decreased the expression of FUBP1 in LoVo almost close to that of LoVo‐shKRAS, while apoptosis inhibitor rescued this inhibition. Silencing FUBP1 substantially reduced the stemness inducing by *KRAS* mutation (Fig. 8B,D). All these data confirmed that FUBP1 was post‐transcriptionally upregulated by caspase 3 inhibition through AKT and ERK activation driven by *KRAS* mutation. The conclusion of the mechanism study fully revealed the reason for the increase in FUBP1 in colorectal cancer, providing another target for treatment (Fig. 8E).

## 4 Discussion

In this study, we revealed that FUBP1 is relevant with tumor progression and metastasis in CRC tissues and cell lines (Figs 1 and 2). Overexpression or knockdown of FUBP1 in CRC cells substantially enhanced or reduced the expression levels of CD133 and ALDH1, the formation of tumor sphere, thus affecting the ability of cell migration and invasion (Figs 2 and 3). Consistently, *in vivo* results demonstrated that overexpression of FUBP1 significantly activated the tumorigenicity of CRC cells (Fig. 4). In terms of mechanism, FUBP1 promoted the initiation of CSCs by directly binding to the *DVL1* promoter to enhance Wnt/β‐catenin signaling. Further, KRAS upregulated FUBP1 through inhibition of caspase 3‐dependent cleavage, as well as the decrease in Smurf2, which promotes ubiquitin‐mediated degradation accounted for the increase in FUBP1 in both *KRAS* wild‐type and mutated CRC patients. This study provides the possibility of a novel treatment strategy for CRC stemness by inhibition of FUBP1‐centered pathway in CRC patients.

FUBP1 could directly bind to the promoter of c‐Myc [10]. Several studies have suggested that c‐Myc played a critical role in promoting stem cell self‐renewal [17] and was reported to promote the CSCs‐like population of CRC cells [23]. Therefore, we need to evaluate the role of c‐Myc in FUBP1‐mediated stemness of CRC. As expected, overexpression of FUBP1 significantly increased c‐Myc expression in CRC cells, while silencing FUBP1 had the reverse effects (Fig. S12E). However, Silencing c‐Myc could not completely reduce FUBP1‐overexpressing cells’ sphere‐forming ability and the expression of stemness‐related markers, CD133, and ALDH1 in FUBP1‐overexpressing CRC cells. Meanwhile, overexpression of c‐Myc failed to wholly rescue the phenotype (Fig. S12F,G), whereas the increased formation of tumor sphere, the abilities of CRC cell migration and invasion, the expression of stemness‐related markers, and Wnt/β‐catenin signaling induced by FUBP1 overexpression were dramatically eliminated by knockdown of DVL1(Fig. 6E,F; Fig. S10B,C). *In vivo* experiments further showed DVL1 was crucial to the tumor volume and tumorigenicity in CDX animal model (Fig. 6G–J). Taken together, elevated FUBP1 promoted CRC cell stemness largely dependent on DVL1 rather than single c‐Myc (Fig. S12).

DVL has been considered as a crucial intermediates of Wnt/β‐catenin signaling pathways, which inhibits GSK‐3β, AXIN, and APC complex formation, regulating cell polarity generation and cell fate specification [24]. APC gene alterations is an early event for 70%–90% of CRC [25]. However, the frequency of abnormal expression of β‐catenin protein far exceeds the frequency of its gene mutation, suggesting other regulatory mechanisms that induced abnormal augmentation of β‐catenin protein in the nucleus of CRC cell [25, 26, 27]. In fact, the misalignment of the APC level is not enough to cause the development of CRC [7]. Other events, such as epigenetic silencing, additional mutations, and microenvironmental signals, are essential for the production of β‐catenin nuclear levels which confer tumorigenic activity [8, 22]. Only the cells with the highest level of Wnt/β‐catenin pathway activation show nuclear localization of β‐catenin with CSC characteristics [8]. In cells where β‐catenin activity is basically dysregulated by APC mutation, other mechanisms can lead to excessive activation of the Wnt/β‐catenin pathway [7].

DVL1 was also reported to be associated with distant metastasis and overall survival in breast cancer patients [28]. Meanwhile, DVL1 was noticeably upregulated in CRC patients with liver metastasis, conferring a poor prognosis [29]. Notably, overexpression of DVL1 in HCC was observed to activate Wnt/β‐catenin signaling, so as to augment tumorigenicity and enhance CSCs‐like phenotype [30]. However, the relationship between FUBP1 and DVL1 had never been reported. Luciferase and ChIP assays confirmed that FUBP1 activated the Wnt/β‐catenin signaling by directly binding to the promoter of *DVL1* in CRC cells (Fig. 6A–D). Moreover, the activation of Wnt/β‐catenin signaling was responsible for the upregulation of pluripotent transcription factors c‐Myc [17], SOX2 [18], and NANOG [19] and stemness induction of CRC cells (Fig. 6E–F; Fig. S5A,B). Our results indicated that the augmented level of FUBP1 promoted the stemness of CRC via enhancing Wnt/β‐catenin signaling.

Collectively, our research first identified that FUBP1 acts as a transcriptional factor for DVL1, and the induction of CRC stemness mainly depends on the activation of the DVL1/Wnt/β‐catenin pathway induced by FUBP1. The highlights of our study are to identify a new target gene of FUBP1 as a transcription factor, in other words, to append a new Wnt‐signaling agonist. This finding provides a novel strategy for the treatment of metastatic CRC by targeting FUBP1 and DVL1. One limitation in the present study is that though one paper designed and generated FUBP1 small molecular inhibitor [31], it is not available for us to test whether this inhibitor can be therapeutically employed in CRC treatment. Since FUBP1 is a transcriptional factor and DVL is the main functional molecule in cytoplasm, we speculate that DVL1 may be more effective and less side effects as a therapeutic target. A specific small‐molecule inhibitor binding to the DVL1 PDZ domain has just been identified and used as a treatment for fibrotic lung disease [20, 32]. It is worth investigating the effect of this specific small‐molecule inhibitor in CRC patients in the following work. Moreover, FUBP1 expression increased in several types of cancers [9], further exploring the role of FUBP1 and DVL1 on stemness transformation and metastasis in those cancers will broaden its significance.

A string of gene mutations characterizes the development of CRC. Among which, *KRAS* mutations are another type of frequent alterations, occurring in 30–50% of CRC cases [33]. *KRAS* mutation has been recently linked to CSCs‐like phenotypes, with functional characteristics of promoting tumor initiation, self‐renewal, and metastasis in CRC cells [22]. Clinical evidence has revealed the association of the poor prognosis and liver metastasis with CSCs of *KRAS* mutation CRC patients [34]. Furthermore, metastasis also occurs in a small proportion of *KRAS* wild‐type patients, which surprisingly has similar CSCs‐like phenotypes in the corresponding cells [35]. Remarkably, the elevated FUBP1 was also observed in about 20% of *KRAS* wild‐type CRC patients with poorer survival outcomes (Fig. 7C,D). These data indicated that FUBP1 possesses the same critical role in *KRAS* wild‐type CRC, and its regulation was independent of *KRAS* mutation. Thus, uncovering the unique mechanism is necessary.

The elevated FUBP1 only occurred in the protein level rather than the mRNA level in CRC, and then, the post‐transcriptionally regulation was mainly focused. Smurf2 is a member of E6‐AP carboxyl terminus E3 ubiquitin ligases family that is important for the ubiquitination of several substrates, including SMAD2, SMAD7, and YY1 [36]. Recent studies reported that Smurf2 was responsible for the ubiquitination of FUBP1, while low expression of Smurf2 was closely relevant to overall survival of CRC patients [21, 37]. Our data revealed that the expression of FUBP1 was negatively associated with Smurf2 in *KRAS* wild‐type patients. Silencing Smurf2 substantially increased the expression of FUBP1 and the stemness‐related markers (Fig. 7G). These results confirmed that FUBP1 was regulated by Smurf2 in CRC. The finding provided insight for the additional treatment of CRC with poor prognosis by upregulating Smurf2, thus recommending Smurf2 expression level as a therapeutic response prediction marker in CRC.

As *KRAS* mutation mediates aberrant ERK and AKT signaling transduction, agents targeted these two pathways were developed. However, targeted therapies by either AKT or ERK pathway inhibitors all failed in the second phase clinical trials [38, 39, 40]. The reason is mainly due to the feedback sensitization of PI3K/AKT signaling after inhibition of RAS/ERK transduction or the drug toxicity of combining ERK and AKT inhibitors [41, 42]. Long‐term studies targeting *KRAS* mutations in cancer have not been successful. In this study, we for the first time reported that FUBP1 functions as a stemness stimulator in CRC. Selectively targeting FUBP1/DVL1, a novel downstream of *KRAS* signaling may be an alternative therapeutic strategy with less toxicity and side effects for *KRAS* mutation CRC.

## 5 Conclusion

In summary, our research demonstrates that elevated FUBP1 in CRC directly upregulates DVL1 and enhances the Wnt/β‐catenin pathway, thereby increasing stemness, promoting metastasis, and conferring a poor prognosis. This study also indicates FUBP1 is a novel and powerful oncogene for the initiation of CSCs and may provide an important prognostic factor and therapeutic target for the efficient elimination of both *KRAS*‐mutant and wild‐type CRC metastasis.

## Author contributions

YX, GGQ, and ZT were responsible for designing and supervising the entire study and revised the manuscript. YHF, GTX, and XJY performed the experiments and analyzed the data. HZJ, ZXY, YFY, QWW, and YZH contributed the data analysis and discussion.

## Conflict of interest

The authors declare no conflict of interest.

## Ethics approval

All procedures are related to animal feeding, treatment, and welfare were conducted in accordance with the Institutional Animal Care and Use Committee of Sun Yat‐sen University.

## Supporting information


**Fig. S1**. LoVo cells exhibit stronger stemness compared with SW48 cells. (A) Western blotting analysis of stemness‐related markers in SW48 and LoVo cells. (B) Flow cytometric analysis proportion of the coexpression of CD133 and ALDH1 in the indicated cells. (C) Representative images of tumor sphere formation in SW48 and LoVo cells. Scale bar, 100μm. (D) Statistical analysis of sphere numbers and sizes in SW48 and LoVo cells. * *P* < 0.05; ** *P* < 0.01. (E) Volcano Plot of differential proteins in CD133^+^ALDH1^+^ LoVo cells versus SW48 cells screened by iTRAQ protein mass spectrometry (log_2_|FC | > 1.2; *P* < 0.005). (F) Venn diagram of proteins related to cell cycle, proliferation, and movement. All bars represented the mean ± SD of three independent experiments. *P* values were determined by two‐tailed Student’s t‐test.

**Fig. S2**. FUBP1 expression was remarkably increased in CRC specimens. (A) Immunohistochemistry staining of FUBP1 expression in a CRC Tissue Microarray (T: Tumor tissue; A: adjacent tissue; HCol‐Ade180Sur‐08‐M‐088). (B) Statistical analysis of cell migration and invasion in the indicated FUBP1‐transfected, FUBP1‐silenced, or vector control cells. ** P < 0.01; *** P < 0.001. All bars represented the mean ± SD of three independent experiments. *P* values were determined by two‐tailed Student’s t‐test.

**Fig. S3**. FUBP1 promotes CRC cell migration and invasion. (A) Representative images of colony formation in the indicated HCT116‐Vector, HCT116‐FUBP1, SW620‐shNC, and SW620‐shFUBP1 cells. (B) Statistical analysis of colony formation. **P < 0.01. (C) Representative images of transwell assays of migration and invasion by the indicated cells. Scale bar, 100μm. (D) Statistical analysis of cell migration and invasion. ** *p* < 0.01; *** *p* < 0.001. (E) Representative images of wound‐healing assays by the indicated cells. (F) Statistical analysis of wound‐healing assays. ** *P* < 0.01; *** *P* < 0.001. All bars represented the mean ± SD of three independent experiments. *P* values were determined by two‐tailed Student’s t‐test.

**Fig. S4**. FUBP1 promotes the stemness of CRC cells *in vitro*. (A) Western blotting analysis of stemness‐related markers, CD133 and ALDH1, in the indicated HCT116‐Vector, HCT116‐FUBP1, SW620‐shNC, and SW620‐shFUBP1 cells. β‐Actin served as a loading control. (B) Flow cytometric analysis proportion of the coexpression of CD133‐PE and ALDH1‐FITC in the indicated cells. (C) Statistical analysis of the proportion of CD133^+^ALDH1^+^ cells. *** *P* < 0.001. (D) Representative images of tumor sphere formation after ten days in nonadherent cultures of the indicated cells. Scale bar, 100μm. (E) Statistical analysis of sphere numbers and sizes after ten days in nonadherent cultures of the indicated cells. * *P* < 0.05; ** *P* < 0.01. All bars represented the mean ± SD of three independent experiments. *P* values were determined by two‐tailed Student’s t‐test.

**Fig. S5**. FUBP1 increases the expression of pluripotent transcription factors. (A, B) The mRNA levels of pluripotent transcription factors by real‐time PCR in FUBP1‐transfected SW48, vector‐transfected SW48, FUBP1‐silenced LoVo, and its control LoVo cells. * *P* < 0.05; ** *P* < 0.01. (C) Luciferase reporter assays of TOP/FOP transcriptional activity in the indicated cells; * *P* < 0.05; ** *P* < 0.01. All bars represented the mean ± SD of three independent experiments. *P* values were determined by two‐tailed Student’s t‐test.

**Fig. S6**. Knockdown of FUBP1 in LoVo spheres decreased the migration and invasion abilities. (A) Representative images of transwell assays of migration and invasion of FUBP1‐silencing LoVo spheres and its control LoVo spheres. Scale bar, 100μm. (B) Statistical analysis of migration and invasion in the indicated cells. ** *p* < 0.01; *** *p* < 0.001. All bars represented the mean ± SD of three independent experiments. *P* values were determined by two‐tailed Student’s t‐test.

**Fig. S7**. The expression of CD133, ALDH1, and DVL1 in Xenograft Models tumors were strongly positively correlated with FUBP1. (A, B) Western blotting (A) and IHC (B) analysis of the expression of CD133, ALDH1, and DVL1 from Xenograft Models tumors. Tumors formed by FUBP1‐transfected SW48, vector‐transfected SW48, FUBP1‐silenced LoVo, and its control LoVo cells.

**Fig. S8**. IHC staining of FUBP1, CD133, and ALDH1 in CRC Tissue Microarrays. (A, B, C) IHC staining of FUBP1, CD133, and ALDH1 in CRC Tissue Microarrays (HCol‐A150CS‐02‐M, 75 cases). Scale bar, 5000μm.

**Fig. S9**. FUBP1 does not affect the receptors and ligands of Wnt/β‐catenin signaling. (A, B) The mRNA levels of receptors and ligands of Wnt/β‐catenin signaling by real‐time PCR in the indicated cells. * *P* < 0.05; *** *P* < 0.001. Bars represented the mean ± SD of three independent experiments. *P* values were determined by two‐tailed Student’s t‐test.

**Fig. S10**. Overexpression of FUBP1 increases the abilities of CRC cell migration and invasion dependent of DVL1. (A) Five truncation fragments of DVL1 promoter. (B) Representative images of migration and invasion transwell assays by the indicated cells. (C) Statistical analysis of cells migration and invasion in the indicated cells. * *P* < 0.05; ** *P* < 0.01. Bars represented the mean ± SD of three independent experiments. *P* values were determined by two‐tailed Student’s t‐test.

**Fig. S11**. FUBP1 binds to DVL's promoter. (A) Statistical analysis of ChIP assays. ** *P* < 0.01; *** *P* < 0.001. (B) Statistical analysis of effects of FUBP1 on tumor sphere formation were blocked after knockdown of DVL1 or recover after overexpression of DVL1. * *P* < 0.05; ** *P* < 0.01; *** *P* < 0.001. All bars represented the mean ± SD of three independent experiments. *P* values were determined by two‐tailed Student’s t‐test.

**Fig. S12**. c‐Myc alone cannot enough promote the stemness of CRC cells. (A) The analysis of FUBP1 mRNA expression of adjacent tissue, tumor in TCGA CRC dataset. (B) The analysis of FUBP1 mRNA expression of SW48 and LoVo cells. (C) Western blotting analysis of FUBP1 expression in the indicated *KRAS* ^WT^ SW48, *KRAS* ^G13D^ SW48, shKRAS LoVo, and its control shNC LoVo cells. β‐Actin served as a loading control. (D) The analysis of FUBP1 mRNA expression of adjacent tissue, *KRAS* wild‐type, and *KRAS* mutation tumor in TCGA CRC dataset. (E) Western blotting analysis of Wnt/β‐catenin signaling and stemness‐related markers in the indicated cells. (F) Representative images of tumor sphere formation by the indicated cells. Scale bar, 50μm. (G) Statistical analysis of sphere numbers and sizes by the indicated cells. * *P* < 0.05; ** *P* < 0.01. All bars represented the mean ± SD of three independent experiments. *P* values were determined by one‐way ANOVA.

**Table S1**. Correlation between expression of FUBP1 and clinicopathological features in 89 cases of CRC.
**Table S2**. Primer sequence.
**Table S3**. Effect of FUBP1 on the tumorigenicity of CRC cell *in vivo* (n = 6/group).
**Table S4**. Effect of FUBP1 on the tumorigenicity of CRC CSCs in vivo (n = 6/group).
**Table S5**. Effect of FUBP1 on the tumorigenicity of SW48 cell *in vivo* (n = 6/group).

## Data Availability

All data generated or analyzed during this study are included in this published article.
