# Supplementary figures and images for: FUBP1 promotes colorectal cancer stemness and metastasis via DVL1‐mediated activation of Wnt/β‐catenin signaling
Source: Mol Oncol. 2021 Jul 29;15(12):3490–512. doi: 10.1002/1878-0261.13064 (PMC8637553; doi:10.1002/1878-0261.13064)

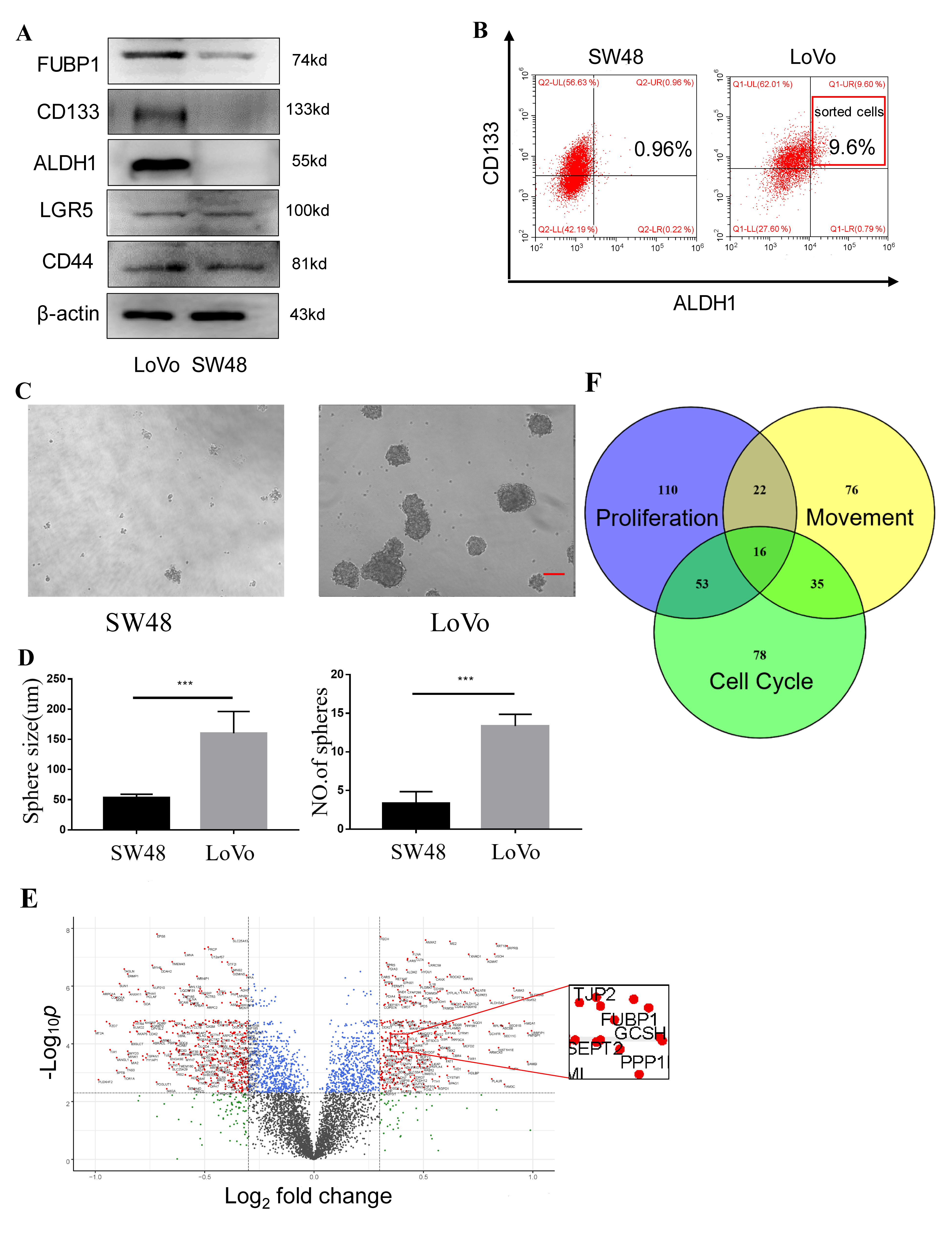

Supplement: Supplementary file 1 — Fig. S1. LoVo cells exhibit stronger stemness compared with SW48 cells. (A) Western blotting analysis of stemness‐related markers in SW48 and LoVo cells. (B) Flow cytometric analysis proportion of the coexpression of CD133 and ALDH1 in the indicated cells. (C) Representative images of tumor sphere formation in SW48 and LoVo cells. Scale bar, 100μm. (D) Statistical analysis of sphere numbers and sizes in SW48 and LoVo cells. * P < 0.05; ** P < 0.01. (E) Volcano Plot of differential proteins in CD133+ALDH1+ LoVo cells versus SW48 cells screened by iTRAQ protein mass spectrometry (log2|FC | > 1.2; P < 0.005). (F) Venn diagram of proteins related to cell cycle, proliferation, and movement. All bars represented the mean ± SD of three independent experiments. P values were determined by two‐tailed Student’s t‐test. [file MOL2-15-3490-s010.png]

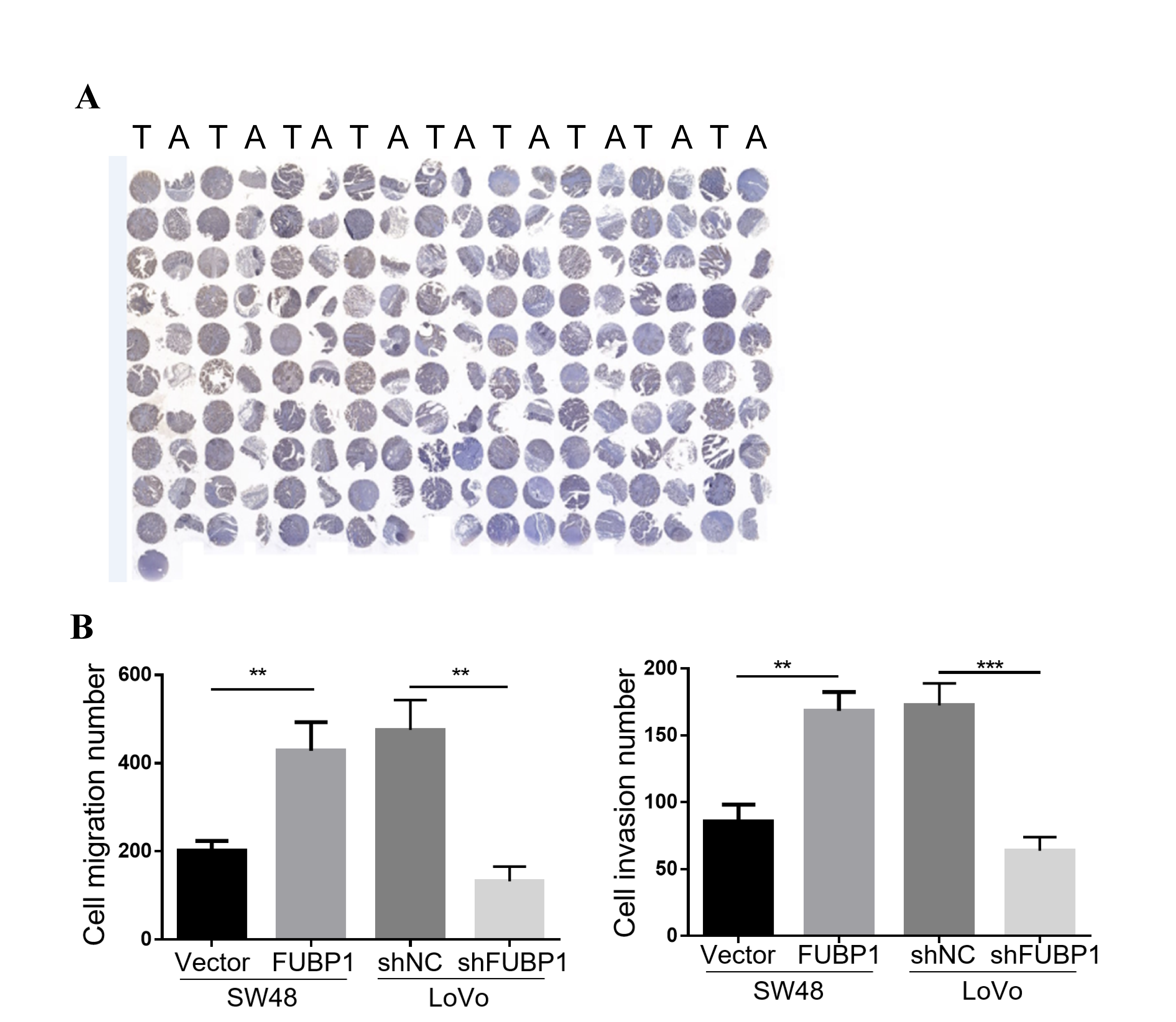

Supplement: Supplementary file 2 — Fig. S2. FUBP1 expression was remarkably increased in CRC specimens. (A) Immunohistochemistry staining of FUBP1 expression in a CRC Tissue Microarray (T: Tumor tissue; A: adjacent tissue; HCol‐Ade180Sur‐08‐M‐088). (B) Statistical analysis of cell migration and invasion in the indicated FUBP1‐transfected, FUBP1‐silenced, or vector control cells. ** P < 0.01; *** P < 0.001. All bars represented the mean ± SD of three independent experiments. P values were determined by two‐tailed Student’s t‐test. [file MOL2-15-3490-s012.png]

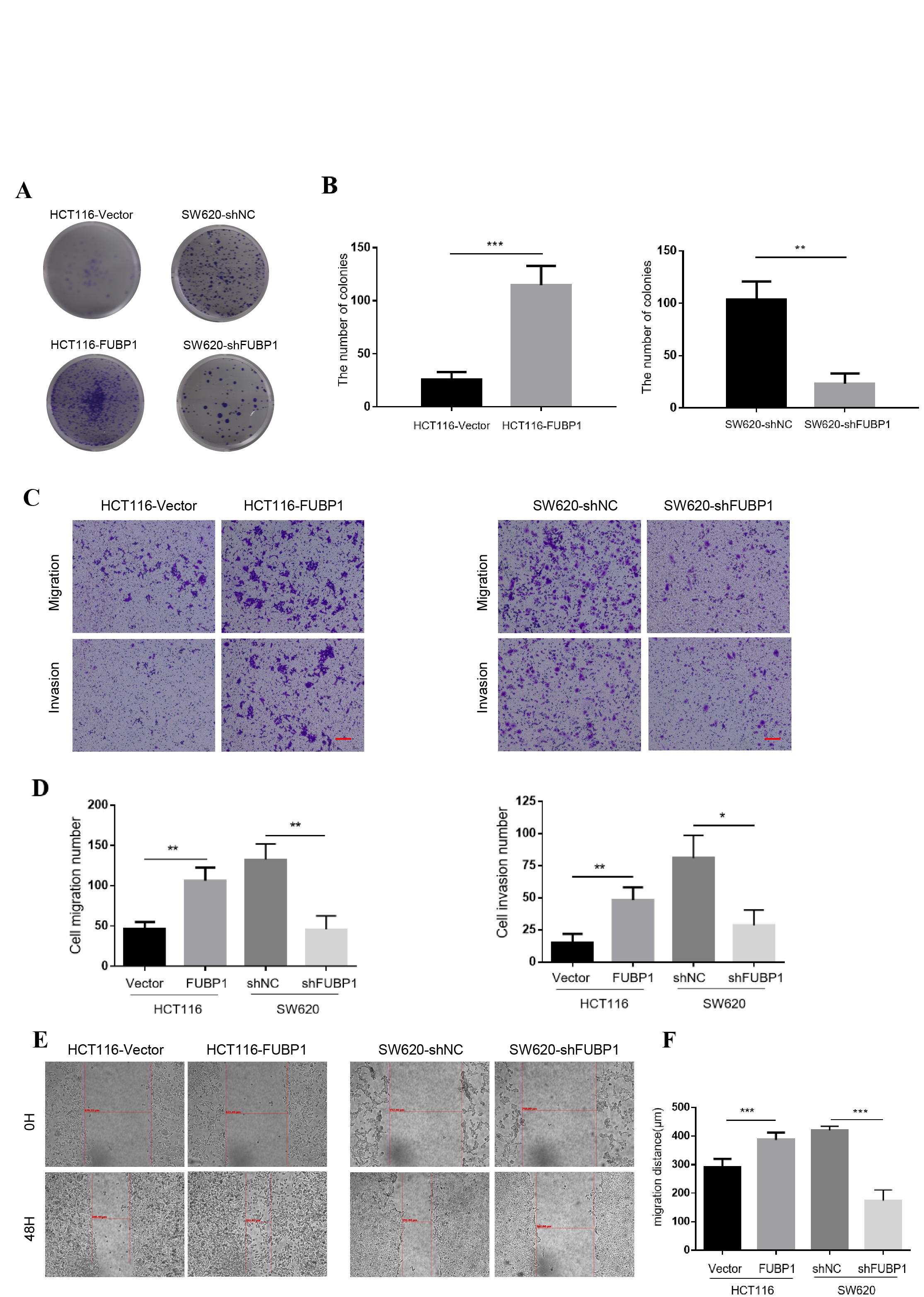

Supplement: Supplementary file 3 — Fig. S3. FUBP1 promotes CRC cell migration and invasion. (A) Representative images of colony formation in the indicated HCT116‐Vector, HCT116‐FUBP1, SW620‐shNC, and SW620‐shFUBP1 cells. (B) Statistical analysis of colony formation. **P < 0.01. (C) Representative images of transwell assays of migration and invasion by the indicated cells. Scale bar, 100μm. (D) Statistical analysis of cell migration and invasion. ** p < 0.01; *** p < 0.001. (E) Representative images of wound‐healing assays by the indicated cells. (F) Statistical analysis of wound‐healing assays. ** P < 0.01; *** P < 0.001. All bars represented the mean ± SD of three independent experiments. P values were determined by two‐tailed Student’s t‐test. [file MOL2-15-3490-s009.tif]

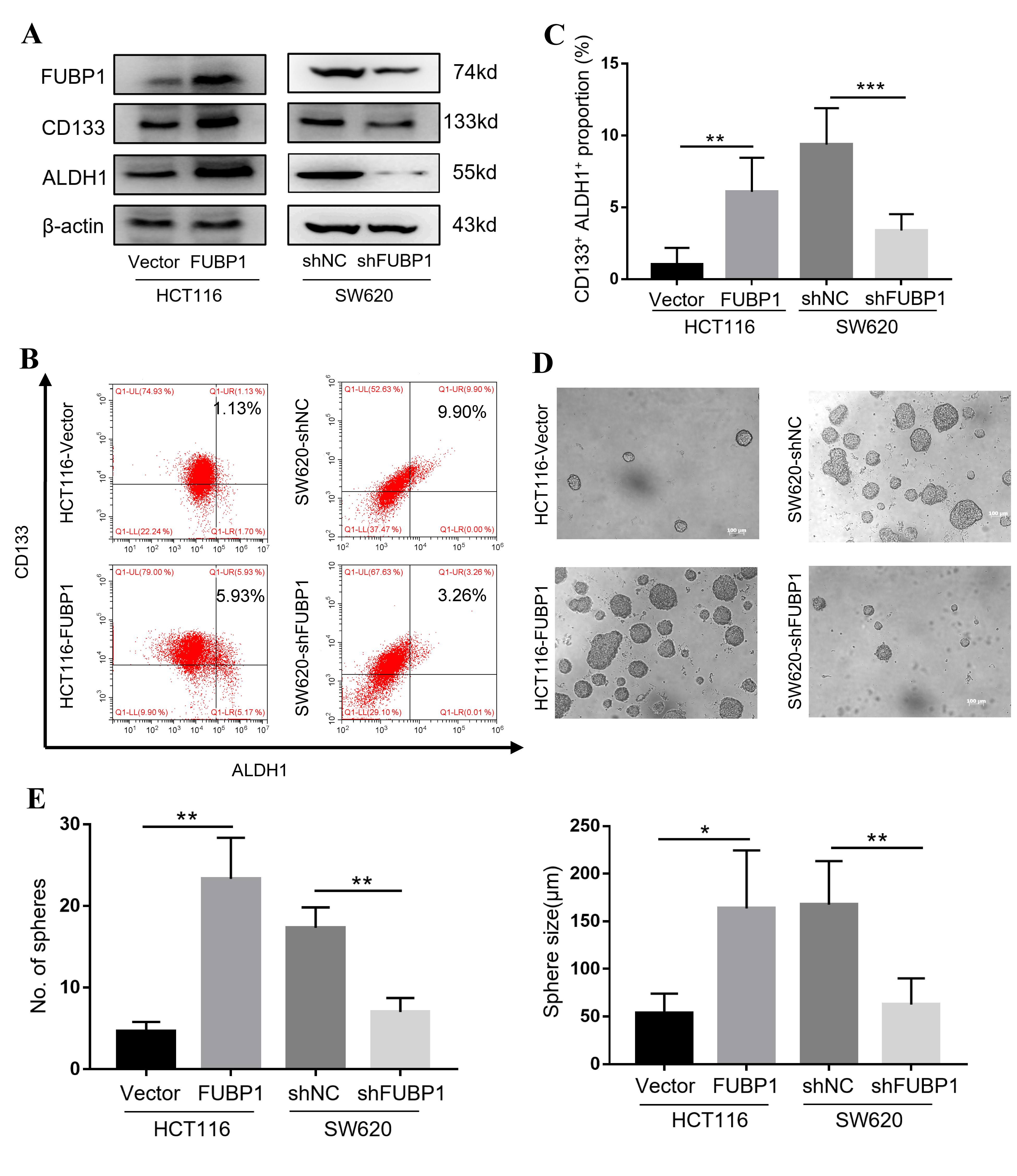

Supplement: Supplementary file 4 — Fig. S4. FUBP1 promotes the stemness of CRC cells in vitro. (A) Western blotting analysis of stemness‐related markers, CD133 and ALDH1, in the indicated HCT116‐Vector, HCT116‐FUBP1, SW620‐shNC, and SW620‐shFUBP1 cells. β‐Actin served as a loading control. (B) Flow cytometric analysis proportion of the coexpression of CD133‐PE and ALDH1‐FITC in the indicated cells. (C) Statistical analysis of the proportion of CD133+ALDH1+ cells. *** P < 0.001. (D) Representative images of tumor sphere formation after ten days in nonadherent cultures of the indicated cells. Scale bar, 100μm. (E) Statistical analysis of sphere numbers and sizes after ten days in nonadherent cultures of the indicated cells. * P < 0.05; ** P < 0.01. All bars represented the mean ± SD of three independent experiments. P values were determined by two‐tailed Student’s t‐test. [file MOL2-15-3490-s005.png]

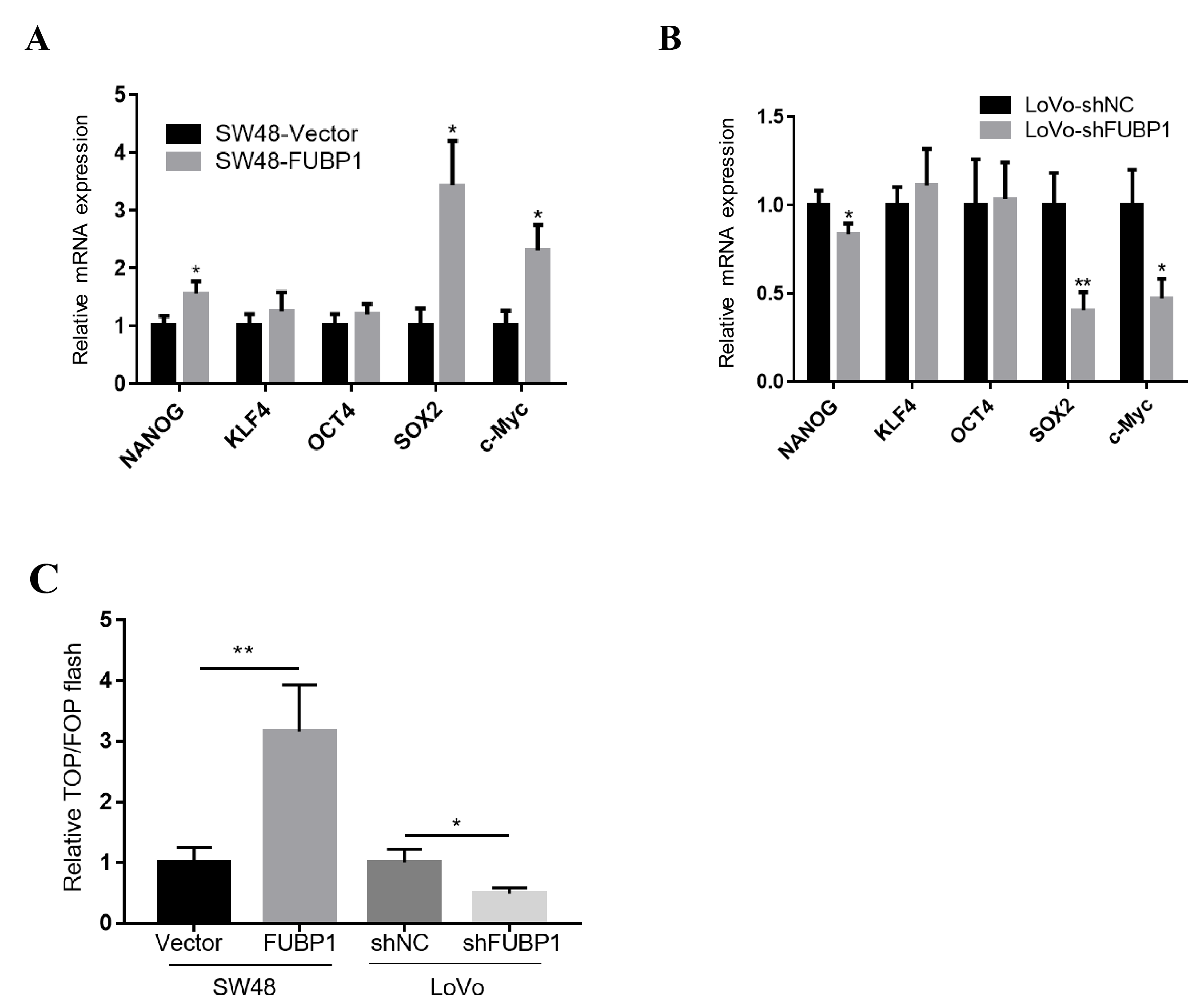

Supplement: Supplementary file 5 — Fig. S5. FUBP1 increases the expression of pluripotent transcription factors. (A, B) The mRNA levels of pluripotent transcription factors by real‐time PCR in FUBP1‐transfected SW48, vector‐transfected SW48, FUBP1‐silenced LoVo, and its control LoVo cells. * P < 0.05; ** P < 0.01. (C) Luciferase reporter assays of TOP/FOP transcriptional activity in the indicated cells; * P < 0.05; ** P < 0.01. All bars represented the mean ± SD of three independent experiments. P values were determined by two‐tailed Student’s t‐test. [file MOL2-15-3490-s011.tif]

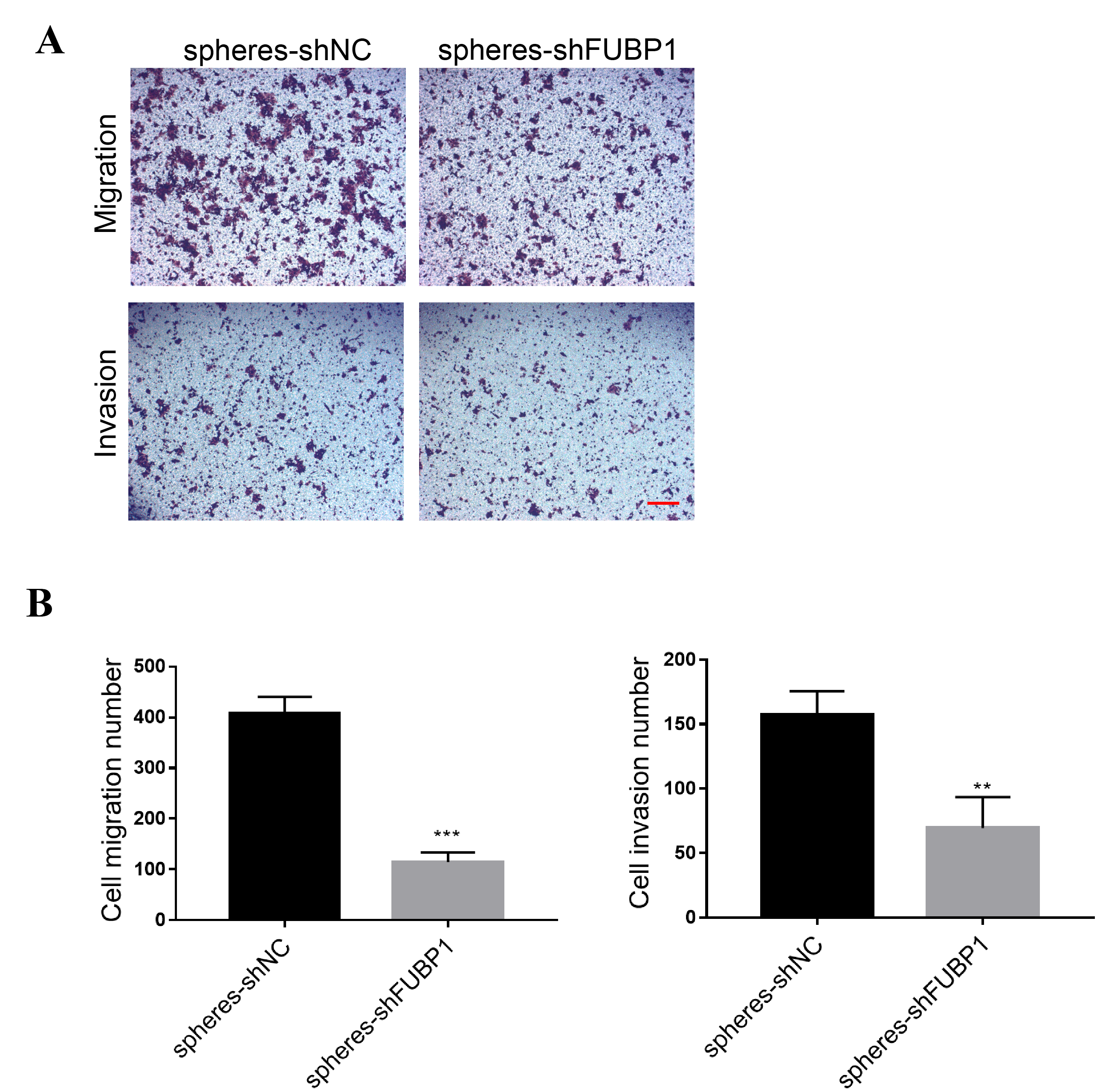

Supplement: Supplementary file 6 — Fig. S6. Knockdown of FUBP1 in LoVo spheres decreased the migration and invasion abilities. (A) Representative images of transwell assays of migration and invasion of FUBP1‐silencing LoVo spheres and its control LoVo spheres. Scale bar, 100μm. (B) Statistical analysis of migration and invasion in the indicated cells. ** p < 0.01; *** p < 0.001. All bars represented the mean ± SD of three independent experiments. P values were determined by two‐tailed Student’s t‐test. [file MOL2-15-3490-s001.tif]

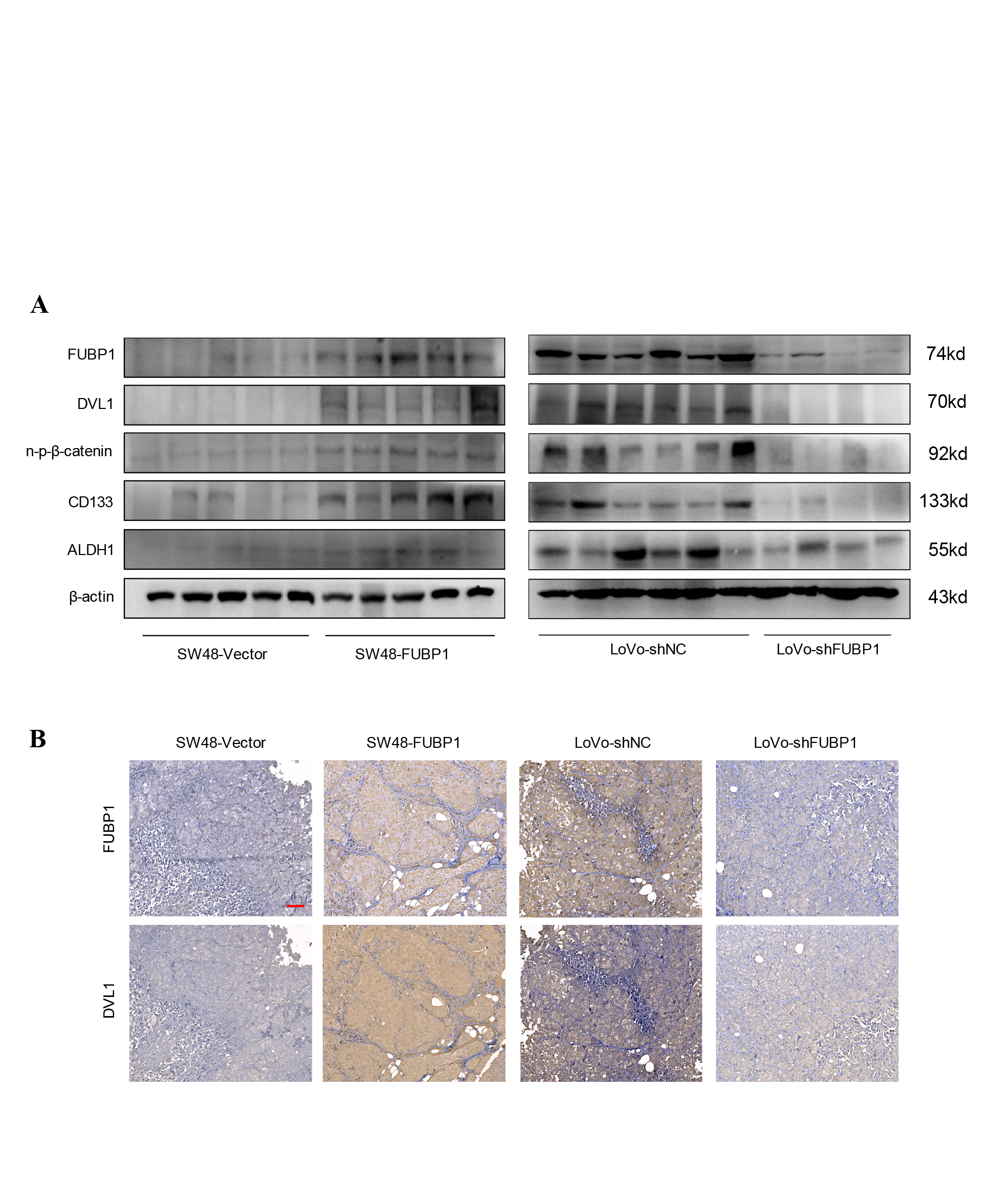

Supplement: Supplementary file 7 — Fig. S7. The expression of CD133, ALDH1, and DVL1 in Xenograft Models tumors were strongly positively correlated with FUBP1. (A, B) Western blotting (A) and IHC (B) analysis of the expression of CD133, ALDH1, and DVL1 from Xenograft Models tumors. Tumors formed by FUBP1‐transfected SW48, vector‐transfected SW48, FUBP1‐silenced LoVo, and its control LoVo cells. [file MOL2-15-3490-s002.tif]

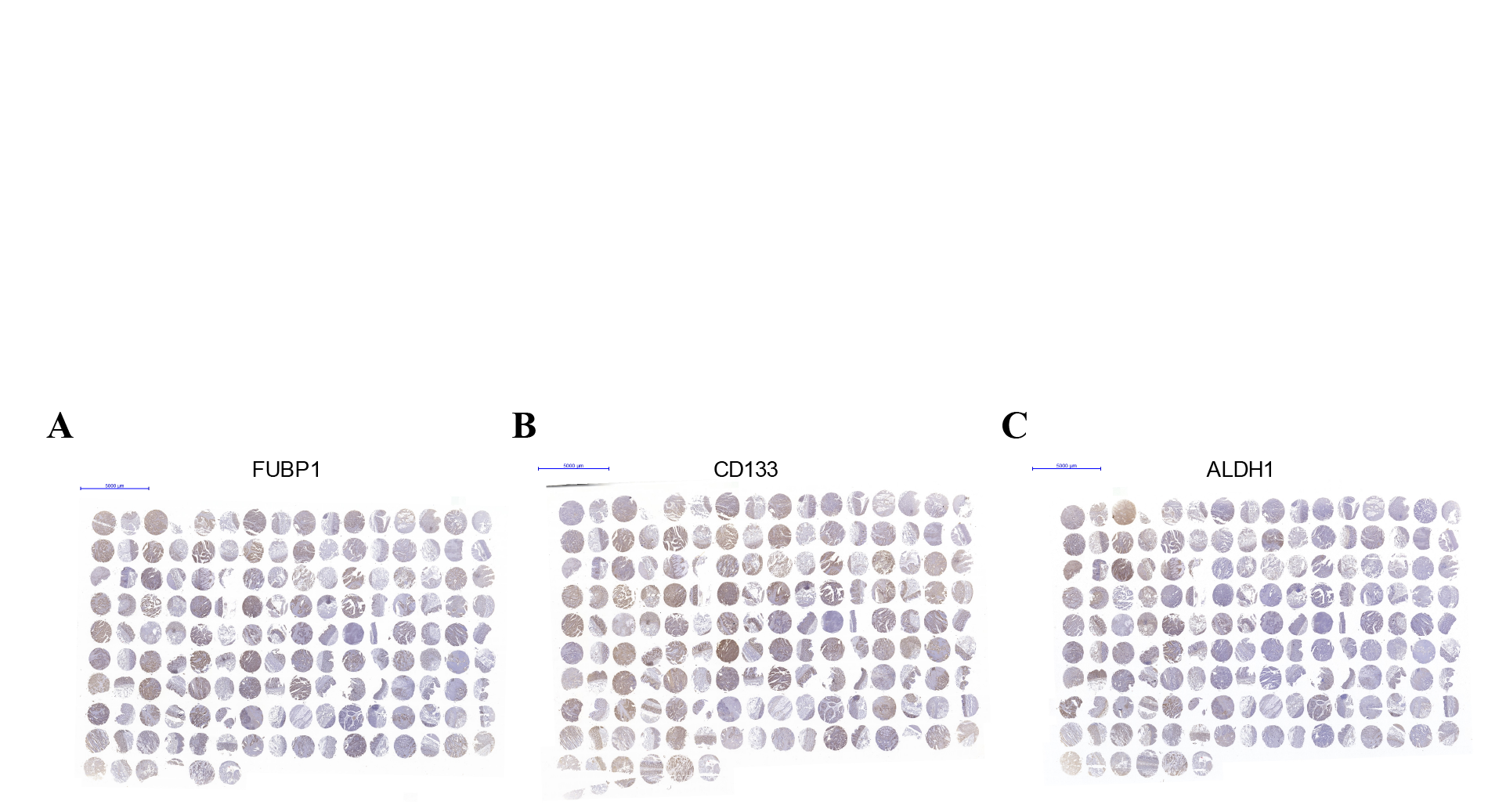

Supplement: Supplementary file 8 — Fig. S8. IHC staining of FUBP1, CD133, and ALDH1 in CRC Tissue Microarrays. (A, B, C) IHC staining of FUBP1, CD133, and ALDH1 in CRC Tissue Microarrays (HCol‐A150CS‐02‐M, 75 cases). Scale bar, 5000μm. [file MOL2-15-3490-s003.tif]

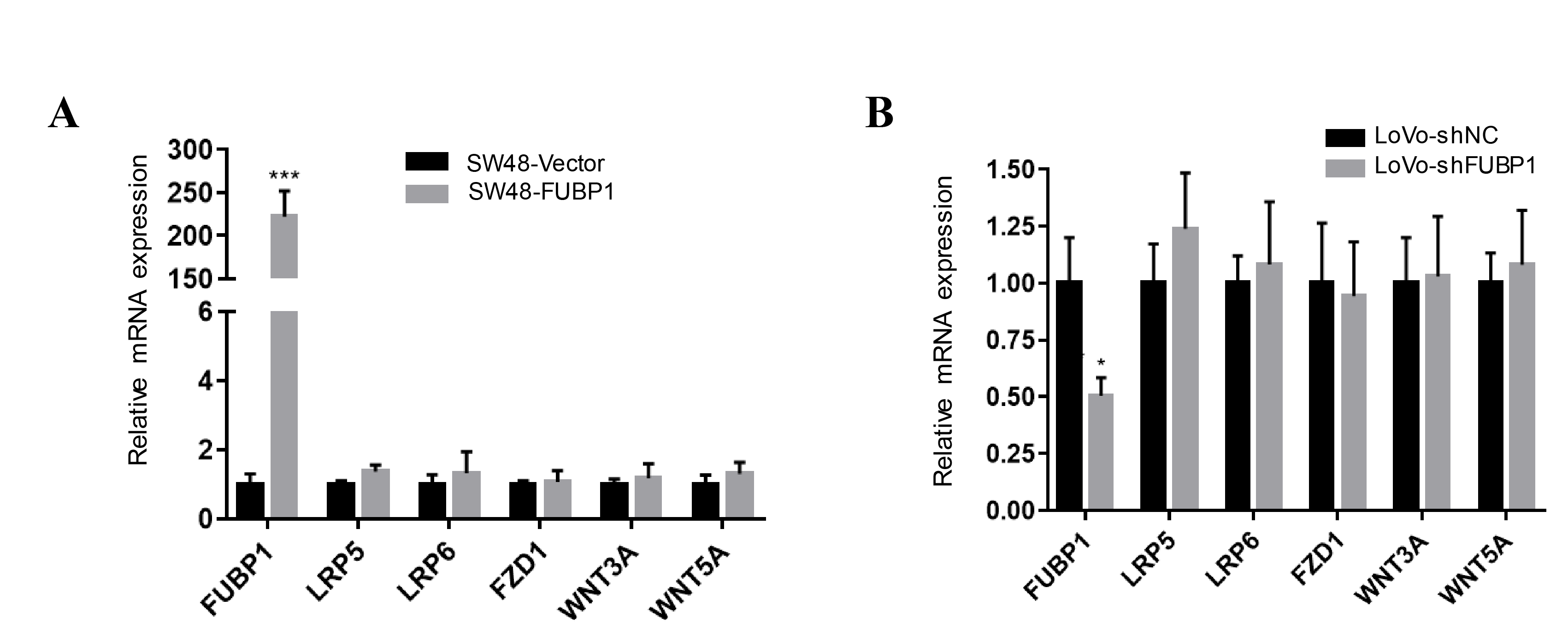

Supplement: Supplementary file 9 — Fig. S9. FUBP1 does not affect the receptors and ligands of Wnt/β‐catenin signaling. (A, B) The mRNA levels of receptors and ligands of Wnt/β‐catenin signaling by real‐time PCR in the indicated cells. * P < 0.05; *** P < 0.001. Bars represented the mean ± SD of three independent experiments. P values were determined by two‐tailed Student’s t‐test. [file MOL2-15-3490-s013.tif]

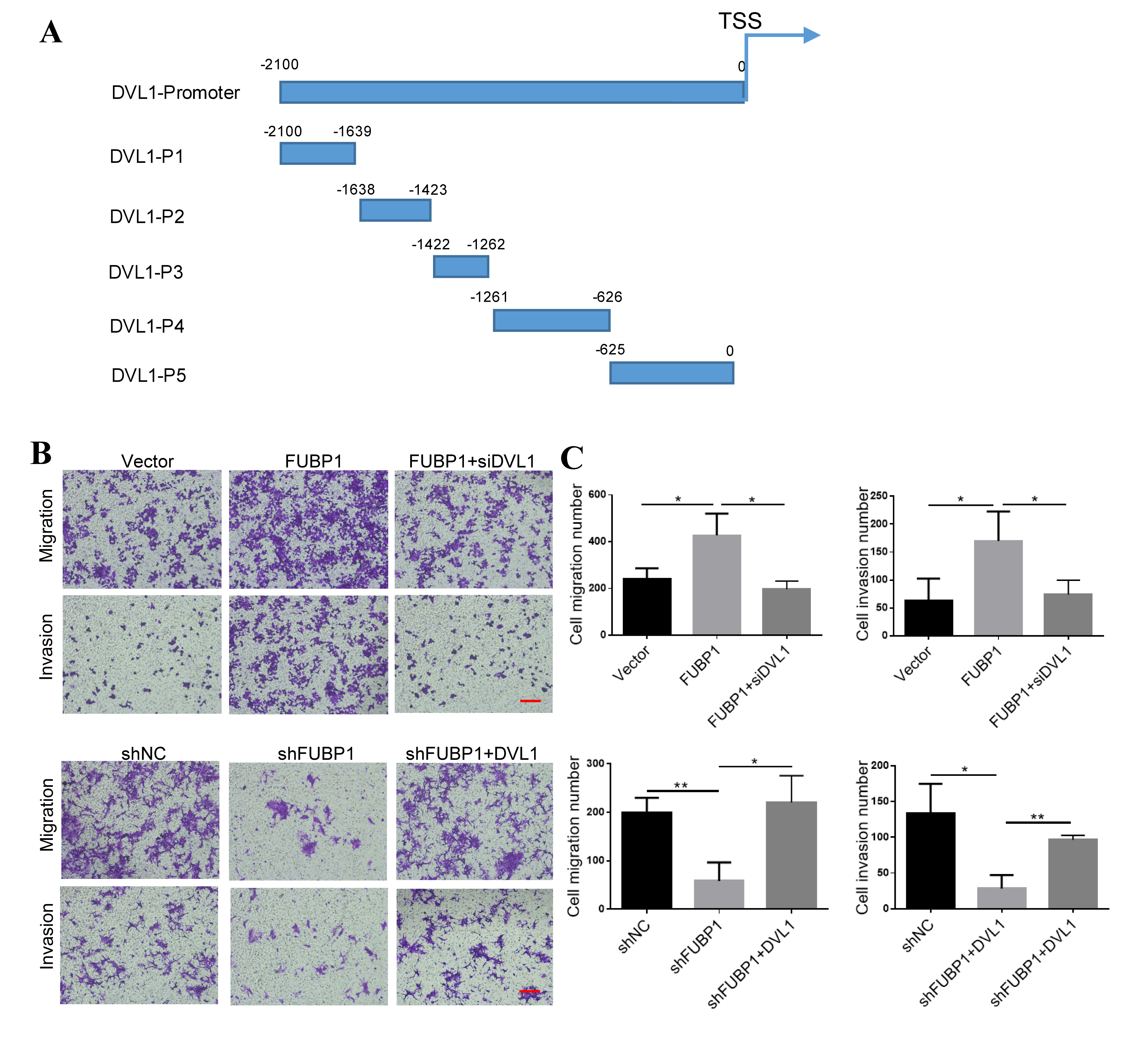

Supplement: Supplementary file 10 — Fig. S10. Overexpression of FUBP1 increases the abilities of CRC cell migration and invasion dependent of DVL1. (A) Five truncation fragments of DVL1 promoter. (B) Representative images of migration and invasion transwell assays by the indicated cells. (C) Statistical analysis of cells migration and invasion in the indicated cells. * P < 0.05; ** P < 0.01. Bars represented the mean ± SD of three independent experiments. P values were determined by two‐tailed Student’s t‐test. [file MOL2-15-3490-s006.png]

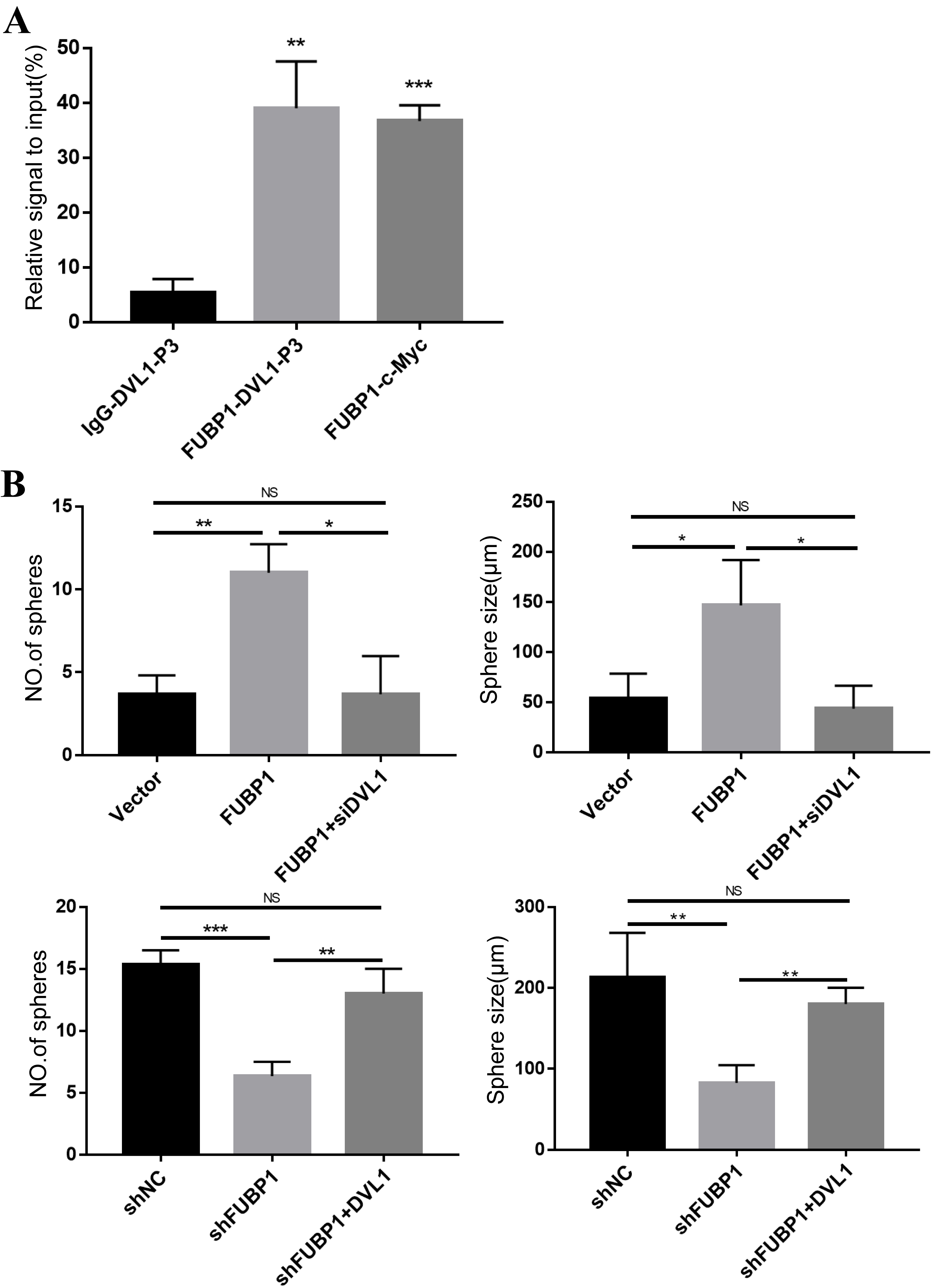

Supplement: Supplementary file 11 — Fig. S11. FUBP1 binds to DVL's promoter. (A) Statistical analysis of ChIP assays. ** P < 0.01; *** P < 0.001. (B) Statistical analysis of effects of FUBP1 on tumor sphere formation were blocked after knockdown of DVL1 or recover after overexpression of DVL1. * P < 0.05; ** P < 0.01; *** P < 0.001. All bars represented the mean ± SD of three independent experiments. P values were determined by two‐tailed Student’s t‐test. [file MOL2-15-3490-s007.png]

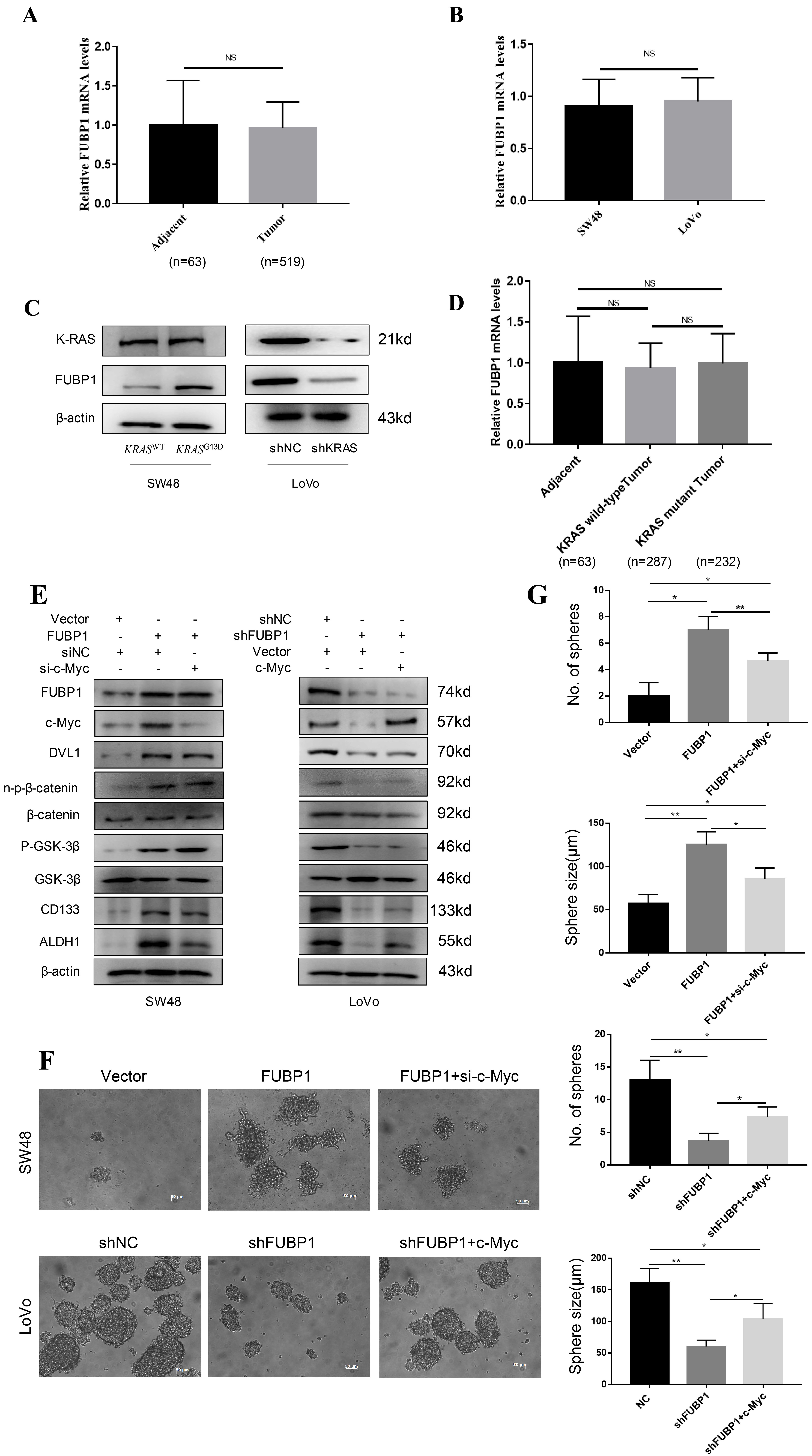

Supplement: Supplementary file 12 — Fig. S12. c‐Myc alone cannot enough promote the stemness of CRC cells. (A) The analysis of FUBP1 mRNA expression of adjacent tissue, tumor in TCGA CRC dataset. (B) The analysis of FUBP1 mRNA expression of SW48 and LoVo cells. (C) Western blotting analysis of FUBP1 expression in the indicated KRAS WT SW48, KRAS G13D SW48, shKRAS LoVo, and its control shNC LoVo cells. β‐Actin served as a loading control. (D) The analysis of FUBP1 mRNA expression of adjacent tissue, KRAS wild‐type, and KRAS mutation tumor in TCGA CRC dataset. (E) Western blotting analysis of Wnt/β‐catenin signaling and stemness‐related markers in the indicated cells. (F) Representative images of tumor sphere formation by the indicated cells. Scale bar, 50μm. (G) Statistical analysis of sphere numbers and sizes by the indicated cells. * P < 0.05; ** P < 0.01. All bars represented the mean ± SD of three independent experiments. P values were determined by one‐way ANOVA. [file MOL2-15-3490-s008.jpg]
