# Supplementary material for: FUBP1 promotes colorectal cancer stemness and metastasis via DVL1‐mediated activation of Wnt/β‐catenin signaling
Source: Mol Oncol. 2021 Jul 29;15(12):3490–512. doi: 10.1002/1878-0261.13064 (PMC8637553; doi:10.1002/1878-0261.13064)
Supplement: Supplementary file 13 — Table S1. Correlation between expression of FUBP1 and clinicopathological features in 89 cases of CRC. Table S2. Primer sequence. Table S3. Effect of FUBP1 on the tumorigenicity of CRC cell in vivo (n = 6/group). Table S4. Effect of FUBP1 on the tumorigenicity of CRC CSCs in vivo (n = 6/group). Table S5. Effect of FUBP1 on the tumorigenicity of SW48 cell in vivo (n = 6/group). [file MOL2-15-3490-s004.docx]

**Supplementary** **Table.1 Correlation between expression of FUBP1 and clinicopathological features in 89 cases of CRC**

| Characteristics |  | No.of patients | | Expression of FUBP1 | | P-value | |
| --- | --- | --- | --- | --- | --- | --- | --- |
|  |  |  |  | Low | High |  |  |
| Patients |  |  |  |  |  |  |  |
| Maligant tumor |  | 89 |  | 43 | 46 | ＜0.001 |  |
| Adjacent tumor |  | 89 |  | 75 | 14 |  |  |
| Age |  |  |  |  |  |  |  |
| ≤60 |  | 21 |  | 10 | 11 | 0.942 |  |
| >60 |  | 68 |  | 33 | 35 |  |  |
| Gender |  |  |  |  |  |  |  |
| Female |  | 44 |  | 22 | 22 | 0.753 |  |
| Male |  | 45 |  | 21 | 24 |  |  |
| Clinical stage |  |  |  |  |  |  |  |
| I+II |  | 56 |  | 32 | 24 | 0.030 |  |
| III+IV |  | 33 |  | 11 | 22 |  |  |
| T classification |  |  |  |  |  |  |  |
| T1-T2 |  | 6 |  | 5 | 1 | 0.075 |  |
| T3-T4 |  | 83 |  | 38 | 45 |  |  |
| N classification |  |  |  |  |  |  |  |
| N0-N1 |  | 80 |  | 42 | 38 | 0.018 |  |
| N2-N3 |  | 9 |  | 1 | 8 |  |  |
| M classification |  |  |  |  |  |  |  |
| M0 |  | 88 |  | 43 | 45 | 0.331 |  |
| M1 |  | 1 |  | 0 | 1 |  |  |
| Differentiation |  |  |  |  |  |  |  |
| Well |  | 23 |  | 16 | 7 | 0.018 |  |
| Moderate/Poor |  | 66 |  | 27 | 39 |  |  |

**Supplementary** **Table.2 Primer sequence**

| Primer | Sequence（5’ to 3’） |
| --- | --- |
| FUBP1-F | TCTTTCTCAGCCCTAACCCA |
| FUBP1-R | CTTGTCCAAGAGCCATCTCCAT |
| DVL1-F | GAGGGTGCTCACTCGGATG |
| DVL1-R | GTGCCTGTCTCGTTGTCCA |
| DVL2-F | GAGGAAGAGACTCCCTACCTG |
| DVL2-R | CGGGCGTTGTCATCTGAAAT |
| DVL3-F | GACGCCGTACCTTGTGAAG |
| DVL3-R | CGCTGCAAAACGCCCTTAAA |
| GSK3β-F | GGCAGCATGAAAGTTAGCAGA |
| GSK3β-R | GGCGACCAGTTCTCCTGAATC |
| APC-F | AAAATGTCCCTCCGTTCTTATGG |
| APC-R | CTGAAGTTGAGCGTAATACCAGT |
| AXIN1-F | GGTTTCCCCTTGGACCTCG |
| AXIN1-R | CCGTCGAAGTCTCACCTTTAATG |
| AXIN2-F | CAACACCAGGCGGAACGAA |
| AXIN2-R | GCCCAATAAGGAGTGTAAGGACT |
| CTNNB1-F | AAAGCGGCTGTTAGTCACTGG |
| CTNNB1-R | CGAGTCATTGCATACTGTCCAT |
| CK1-F | AGTGGCAGTGAAGCTAGAATCT |
| CK1-R | CGCCCAATACCCATTAGGAAGTT |
| SOX2-F | GCCGAGTGGAAACTTTTGTCG |
| SOX2-R | GGCAGCGTGTACTTATCCTTCT |
| c-Myc-F | GGCTCCTGGCAAAAGGTCA |
| c-Myc-R | CTGCGTAGTTGTGCTGATGT |
| NANOG-F | TTTGTGGGCCTGAAGAAAACT |
| NANOG-R | AGGGCTGTCCTGAATAAGCAG |
| KLF4-F | CCCACATGAAGCGACTTCCC |
| KLF4-R | CAGGTCCAGGAGATCGTTGAA |
| OCT4-F | CTGGGTTGATCCTCGGACCT |
| OCT4-R | CCATCGGAGTTGCTCTCCA |
| COX2-F | CTGGCGCTCAGCCATACAG |
| COX2-R | CGCACTTATACTGGTCAAATCCC |
| MMP7-F | GAGTGAGCTACAGTGGGAACA |
| MMP7-R | CTATGACGCGGGAGTTTAACAT |
| CCND1-F | GCTGCGAAGTGGAAACCATC |
| CCND1-R | CCTCCTTCTGCACACATTTGAA |
| β-actin-F | ACTCTTCCAGCCTTCCTTC |
| β-actin-R | ATCTCCTTCTGCATCCTGTC |
| CHIP-DVL1-3-F | GTCAGGGTCAGGAGACAACC |
| CHIP-DVL1-3-R | GCCAGAATGACGGGAACA |
| CHIP-c-Myc-F | CCCCCGAATTGTTTTCTCTT |
| CHIP-c-Myc-R | CCCACACATGATTTGTTTGC |

**Supplementary** **Table.3 Effect of FUBP1 on the tumorigenicity of CRC cell *in vivo* (n = 6/group).**

| Cell type | Cell number | Tumor incidence | TIC frequency | *P* value |
| --- | --- | --- | --- | --- |
| SW48-Vector  SW48-FUBP1  LoVo-shNC  LoVo-shFUBP1 | 10^6^  10^5^  10^4^  10^3^  10^6^  10^5^  10^4^  10^3^  10^6^  10^5^  10^4^  10^3^  10^6^  10^5^  10^4^  10^3^ | 5/6  3/6  2/6  1/6  6/6  5/6  3/6  2/6  6/6  5/6  4/6  2/6  4/6  3/6  2/6  1/6 | 1/236764  (1/638694-1/87769)  1/79188  (1/71233-1/10397)  1/22860  (1/62367-1/8379)  1/368950  (1/908299-1/149867) | ＜0.001  ＜0.001 |

**Supplementary** **Table.4 Effect of FUBP1 on the tumorigenicity of CRC CSCs in vivo (n = 6/group).**

| Cell type | Cell number | | Tumor incidence | TIC frequency | *P* value |
| --- | --- | --- | --- | --- | --- |
| CSCs-shNC  CSCs-shFUBP1 | | 10^5^  10^4^  10^3^  10^2^  10^5^  10^4^  10^3^  10^2^ | 5/6  5/6  4/6  3/6  3/6  2/6  1/6  0/6 | 1/11309  (1/30399-1/4207)  1/79188  (1/198124-1/31650) | ＜0.001 |

**Supplementary** **Table.5 Effect of FUBP1 on the tumorigenicity of SW48 cell *in vivo* (n = 6/group).**

| Cell type | Cell number | Tumor incidence | TIC frequency |  |
| --- | --- | --- | --- | --- |
| SW48-Vector  SW48-FUBP1  SW48-FUBP1+shDVL1  SW48-FUBP1+NSC668036 | 10^5^  10^4^  10^3^  10^5^  10^4^  10^3^  10^5^  10^4^  10^3^  10^5^  10^4^  10^3^ | 2/6  2/6  1/6  4/6  3/6  2/6  3/6  2/6  1/6  3/6  1/6  1/6 | 1/108027  (1/289255-1/40344)  1/41820  (1/101669-1/17202)  1/79081  (1/197964-1/31591)  1/97018  (1/252927-1/37214) |  |

**Supplementary** **Table.6 Effect of FUBP1 on the tumorigenicity of LoVo cell *in vivo* (n = 6/group).**

| Cell type | Cell number | Tumor incidence | TIC frequency |  |
| --- | --- | --- | --- | --- |
| LoVo-shNC  LoVo-shFUBP1  LoVo-shFUBP1+DVL1 | 10^5^  10^4^  10^3^  10^5^  10^4^  10^3^  10^5^  10^4^  10^3^ | 5/6  3/6  2/6  3/6  1/6  1/6  4/6  3/6  2/6 | 1/27214  (1/71233-1/10397)  1/97018  (1/252927-1/37214)  1/41820  (1/101669-1/17202) |  |
